# Targeting TRIM15-mediated Axin1 depolymerization suppresses Wnt signaling and inhibits colorectal cancer growth

**DOI:** 10.1038/s41419-025-08400-7

**Published:** 2025-12-29

**Authors:** Hangfei Liang, Fanghong Zheng, Jincheng Wu, Han Zhou, Zhouyi Sun, Pengfei Zhang, Wei Wu, Guixin Zhu

**Affiliations:** 1 Department of General Surgery, Center for Oncology Medicine, the Fourth Affiliated Hospital of School of Medicine, and International School of Medicine, International Institutes of Medicine, Zhejiang University, Yiwu, China; 2 Zhejiang Key Laboratory of Precision Diagnosis and Treatment for Lung Cancer, International Institutes of Medicine, Zhejiang University, Yiwu, China; 3 MOE Key Laboratory of Protein Sciences, Beijing Advanced Innovation Center for Structural Biology, School of Life Sciences, Tsinghua University, Beijing, China; 4 Institutes of Biomedical Sciences, Inner Mongolia University, Hohhot, China; 5 School of Molecular Medicine, Hangzhou Institute for Advanced Study, University of Chinese Academy of Sciences, Hangzhou, China; 6 University of Chinese Academy of Sciences, Beijing, China

**Keywords:** Cell signalling, Gastrointestinal cancer

## Abstract

Axin1 plays a critical role in regulating the Wnt/β-catenin signaling pathway and cancer progression, and its polymerization is indispensable for the assembly of the β-catenin destruction complex. However, the mechanisms that control Axin1 polymerization are limited. Here, we reveal that TRIM15 interferes with the polymerization of Axin1, thereby promoting Wnt activation and colorectal cancer growth. Mechanistically, TRIM15 strongly interacts with Axin1 through its coiled-coil domain to disrupt the polymerization among Axin1 molecules. Manipulation of TRIM15 expression dramatically weakens Wnt signaling, cell proliferation, and tumor growth. Furthermore, conditional genetic ablation of *Trim15* in mice inhibits tumor formation in both AOM/DSS-induced and *Apc*^Min/+^ colorectal cancer models. Notably, *TRIM15* is also a Wnt target gene that forms a positive feedback loop in colon cancer cells. TRIM15 is highly expressed and is positively associated with β-catenin in colorectal cancer. More importantly, the simultaneous increase in Axin1 protein levels and its polymerization can synergistically induce apoptosis. Together, our study uncovers an important regulatory mechanism of Axin1 polymerization and implies that targeting TRIM15 provides a therapeutic strategy for colorectal cancer based on inhibiting Wnt signaling.

## Introduction

Colorectal cancer (CRC), a kind of lethal malignant tumor, currently ranks third in the global incidence spectrum of tumors [1]. The onset of CRC is a complicated and multilayered process that involves the accumulation of a series of mutated oncogenes and tumor suppressor genes [2]. *APC*, as a tumor suppressor gene, frequently leads to the formation of polyps in the colon and rectum upon mutation. With more mutations in certain genes, the polyps may subsequently undergo malignant transformation into CRC [3–5]. Throughout the entire process, the Wnt/β-catenin signaling is gradually activated [5, 6]. Accumulating evidence shows that Wnt/β-catenin signaling is tightly associated with colorectal tumorigenesis [7–9]. Targeting Wnt/β-catenin signaling may be a promising strategy towards CRC [7, 10].

Wnt/β-catenin signaling, also known as canonical Wnt signaling, is evolutionarily conserved across different species [11]. In the absence of Wnt activation, cytoplasmic β-catenin is recruited to the β-catenin destruction complex, primarily consisting of Axin1, APC, GSK3β, and CK1, to undergo phosphorylation and subsequent degradation via SCF^β-TrCP^-mediated proteasome degradation. Upon Wnt activation, the β-catenin destruction complex is disrupted, resulting in the stabilization of β-catenin. This stabilized β-catenin enters the nucleus, where it co-opts with TCF/LEF transcriptional factors to promote the transcription of downstream target genes, such as *CCND1*, *MYC* [12–15]. Thus, the assembly and disassembly of the β-catenin destruction complex are crucial for Wnt signaling activation.

Axin1, a key negative regulator of Wnt signaling, is considered a rate-limiting factor within the β-catenin destruction complex, which is attributed to its much lower protein level compared to other complex components [16, 17]. Moreover, Axin1 serves as a scaffold protein to bind to all components within the destruction complex, enabling rapid and efficient assembly of the complex, which facilitates the phosphorylation and ultimate degradation of β-catenin [16, 18]. Of note, the interaction between Axin1 molecules leads to polymerization, which acts as a major determinant in the assembly and size of the β-catenin destruction complex [16]. The polymerization of Axin1 can more effectively recruit other components to assemble the destruction complex to further enhance phosphorylation and degradation of β-catenin. Therefore, the interference of Axin1 polymerization significantly impairs the ability of the destruction complex to phosphorylate and degrade β-catenin. However, there is limited understanding regarding how Axin1 polymerization is regulated.

TRIM15, as a member of the TRIM (tripartite motif) family proteins, plays important roles in several kinds of cancer through ubiquitination [19–21], while its functions and biological significance in colorectal cancer growth remain unknown. In this study, we discovered that TRIM15 interacts with Axin1 and controls its polymerization but not stabilization, thereby promoting Wnt/β-catenin activation to support colorectal cancer growth. Meanwhile, we also identified that *TRIM15* is a Wnt target gene in colorectal cancer cell lines. Hence, our study describes a mechanism of reciprocal promotion of TRIM15 and Wnt/β-catenin signaling, providing a potential therapeutic target for colorectal cancer treatment.

## Results

### TRIM15 interacts with Axin1

**Fig. 1:**
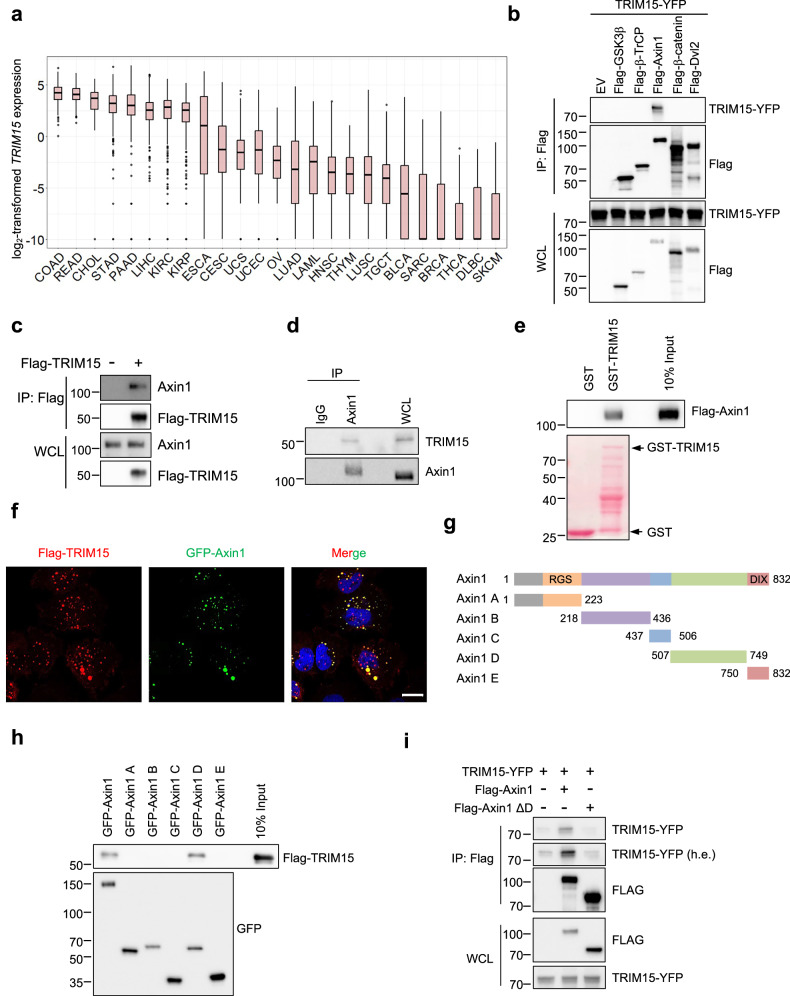
E3 ubiquitin ligase TRIM15 specifically interacts with Axin1. **a** Pan-cancer analysis of TRIM15 expression. mRNA expression levels (log2(TPM + 1)) of TRIM15 across different tumor types from The Cancer Genome Atlas (TCGA) were shown as box plots, with the midline representing the mean value. **b** HEK293T cells transfected with the indicated plasmids were subjected to Co-IP. IP samples and whole cell lysates (WCL) were analyzed by Western blot. **c** Co-IP analysis of the interaction between Flag-TRIM15 and endogenous Axin1 in HCT116 cells. IP samples and WCL were analyzed by Western blot. **d** Cell lysates derived from HCT116 cells were analyzed by co-IP with control IgG or anti-Axin1 antibody. IP samples and WCL were analyzed by Western blot. **e** Flag-Axin1, purified from HEK293T cells, was incubated with either immobilized GST or GST-TRIM15, both of which were purified from bacteria. The pulldown samples and input were analyzed by SDS–PAGE followed by Western blot and/or Ponceau S staining. **f** Immunofluorescence staining of Flag-TRIM15 and GFP-Axin1 in HeLa cells. Nuclei (blue) were counterstained with DAPI. Scale bar represents 20 μm. **g** Schematic illustration of full-length and truncated Axin1. **h** Flag-TRIM15, purified from HEK293T cells, was incubated with immobilized GFP-fused Axin1 or its fragments, all of which were also purified from HEK293T cells. The pulldown samples and input were analyzed by SDS–PAGE followed by Western blot. **i** HEK293T cells transfected with TRIM15-YFP along with either Flag-Axin1 or Flag-Axin1 ΔD plasmids were subjected to Co-IP using anti-Flag beads. IP samples and WCL were analyzed by Western blot.

Our previous data demonstrated that TRIM15, as a ubiquitin E3 ligase, contributes to melanoma progression through direct ubiquitination and activation of ERK1/2 [19]. However, its role in colorectal cancer remains elusive. Surprisingly, among many kinds of tumor types, the expression level of TRIM15 is the highest in colon and rectal cancer patients (Fig. 1a; Extended Data Fig. S1a and b). Consistent with this phenomenon, TRIM15 is also expressed, with the highest levels in colorectal cancer cell lines by analysis of the HPA database (Extended Data Fig. S1c). Wnt/β-catenin signaling plays a critical role in colorectal cancer progression. These prompted us to test whether TRIM15 has the potential to bind to proteins that have vital functions in Wnt signaling. As shown in Fig. 1b and Extended Data S1d, exogenous TRIM15 specifically interacts with exogenous Axin1, a critical component in regulating the assembly of the destruction complex [16]. Moreover, TRIM15 also interacted with Axin1 endogenously (Fig. 1c, d). This was further confirmed by GST-pulldown assay, indicating a direct interaction between TRIM15 and Axin1 (Fig. 1e). Immunofluorescence staining showed that TRIM15 was co-localized with Axin1, with both exhibiting dot-like structures (Fig. 1f).

It is well established that the DIX domain of Axin1 is required for its polymerization, which is necessary for forming dot-like structures [22, 23]. We first investigated whether DIX mediated the affinity of Axin1 with TRIM15. Unexpectedly, depletion of the DIX domain in Axin1 did not affect the interaction between Axin1 and TRIM15 (Extended Data Fig. S1e). According to the distinct functional domains of Axin1 [24] (Fig. 1g), we generated a series of its truncated mutants and performed co-immunoprecipitation (Co-IP) analyses. As shown in Fig. 1h, Axin1 D domain specifically pulled down TRIM15. Additionally, Axin1 without D domain failed to associate with TRIM15 (Fig. 1i), indicating that Axin1 D domain is required for binding to TRIM15. Taken together, these results demonstrate that Axin1 interacts with TRIM15 through its D domain, but not through its DIX domain.

### TRIM15 coiled coil domain is essential for binding to Axin1

**Fig. 2:**
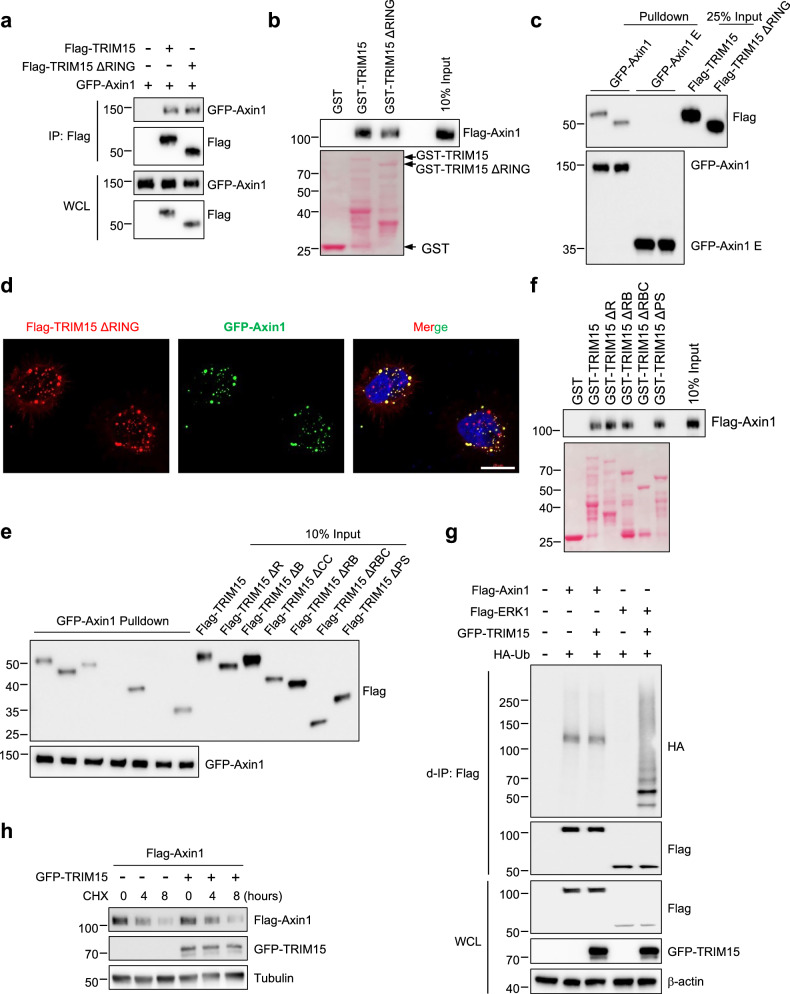
TRIM15 Coiled coil domain is required for binding to Axin1. **a** HEK293T cells transfected with the indicated plasmids were subjected to Co-IP using anti-Flag beads. IP samples and WCL were analyzed by Western blot. **b** Flag-Axin1, purified from HEK293T cells, was incubated with either immobilized GST or GST-fused TRIM15 proteins, all of which were purified from bacteria. The pulldown samples and input were analyzed by Western blot and/or Ponceau S staining. **c** Purified Flag-TRIM15 or Flag-TRIM15 ΔRING proteins were incubated with immobilized GFP-fused Axin1 or E fragment. The pulldown and input samples were analyzed by Western blot. **d** Immunofluorescence staining of Flag-TRIM15 ΔRING and GFP-Axin1 in HeLa cells. Nuclei (blue) were counterstained with DAPI. Scale bar represents 20 μm. **e** Purified Flag-TRIM15 or its deletion proteins were incubated with immobilized GFP-Axin1. The pulldown samples and input were analyzed by Western blot. **f** Purified Flag-Axin1 was incubated with immobilized GST, GST-fused TRIM15, or its deletion proteins. The pulldown and input samples were analyzed by Western blot and/or Ponceau S staining. **g** Cell lysates from HEK293T cells transfected with the indicated plasmids were boiled in SDS-containing buffer, diluted, and d-IP using anti-Flag beads. IP samples and WCL were analyzed by Western blot. **h** HEK293T cells transfected with Flag-Axin1 with or without GFP-TRIM15. Protein stability was examined by collecting samples at the indicated time after CHX (100 μg/mL) treatment.

Since TRIM15 is a ubiquitin E3 ligase, we reasonably predicted that the RING domain of TRIM15 might be involved in regulating its interaction with Axin1. However, TRIM15 still retained the ability to interact with Axin1 when RING domain was deleted (Fig. 2a). GST-pulldown assay further confirmed the phenomenon that the RING domain was not directly engaged in association with Axin1 (Fig. 2b). Similarity, by using GFP-fused Axin1 or E (DIX domain) beads, in vitro pulldown assays were carried out to uncover that TRIM15 interacted with Axin1 independent of its RING domain (Fig. 2c). Besides, immunofluorescence staining delineated that TRIM15 ∆RING was still co-localized with Axin1 (Fig. 2d). These data suggested that TRIM15 RING domain was not required for binding to Axin1, which implies a non-catalytic function of TRIM15 for Axin1 regulation.

To explore the key domain of TRIM15 required for affinity with Axin1, a panel of TRIM15 deletion mutants was generated (Extended Data Fig. S2a). We used these mutants to perform Co-IP analyses in HEK293T cells, which revealed that TRIM15 lacking the CC domain lost its ability to interact with Axin1 (Extended Data Fig. S2b). In addition, the vitro pulldown assay exhibited that the CC domain of TRIM15 was eminently important, because TRIM15 mutants without CC domain (∆CC and ∆RBC) was unable to interact with Axin1 (Fig. 2e). GST-TRIM15 truncated proteins purified from E. coli were also capable of associating with Axin1 except the protein without CC domain (Fig. 2f). These data explicitly indicated that the CC domain of TRIM15 is essential for interacting with Axin1. Next, we sought to determine whether TRIM15 affects Axin1 stability. Denatured-IP (d-IP) assay revealed that TRIM15 could promote ERK1 ubiquitination (used as a positive control) but not Axin1 ubiquitination (Fig. 2g), suggesting that TRIM15 probably does not regulate the stability of Axin1 through ubiquitination. Indeed, in a protein half-life assay, Axin1 degradation was unaffected by the overexpression of TRIM15 (Fig. 2h). Overall, these findings show that TRIM15 interacts with Axin1 through its CC domain and exerts little effect on Axin1 protein stability, despite being an E3 ubiquitin ligase.

### TRIM15 regulates Axin1 polymerization and β-catenin destruction complex assembly

**Fig. 3:**
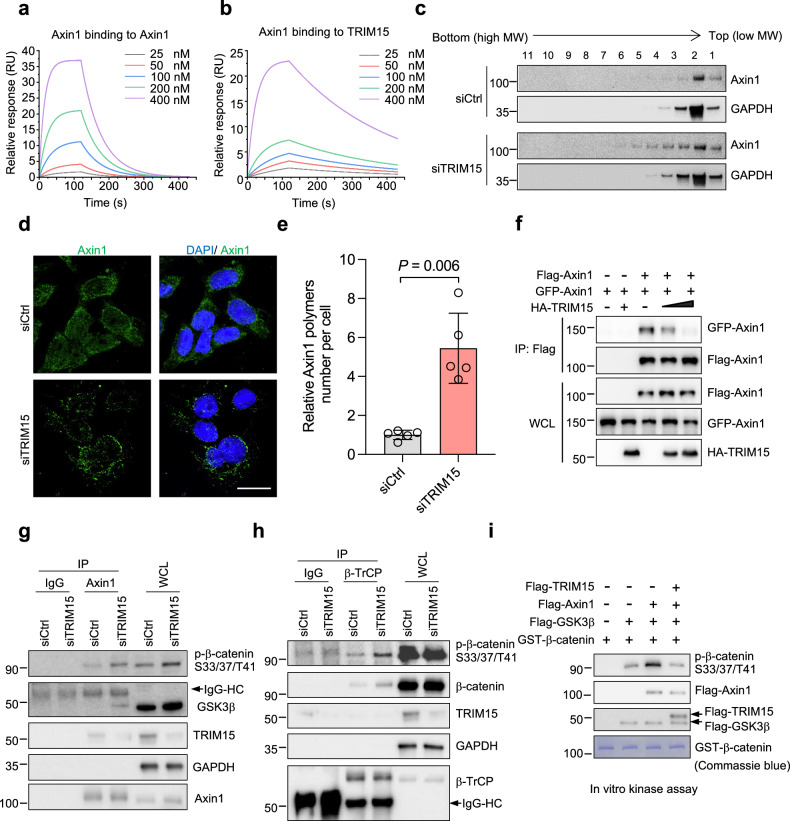
TRIM15 controls Axin1 polymerization and β-catenin destruction complex assembly. SPR (BIAcore) analysis of the dissociation constant (*K*_d_) for Axin1 (**a**) or TRIM15 (**b**) from immobilized Axin1. Axin1 and Axin1 interaction had a calculated *K*_d_ of 147 nM, Axin1 and TRIM15 interaction had a calculated *K*_d_ of 37.8 nM. **c** HCT116 cells were transfected with control siRNA (siCtrl) or TRIM15 siRNA (siTRIM15), followed by a sucrose density gradient centrifugation experiment to assess the polymerization degree of Axin1 protein. Immunofluorescence staining of Axin1 in DLD-1 cells transfected with siCtrl or TRIM15 siTRIM15 for 48 h. Representative confocal images of endogenous Axin1 (**d**) and the number of Axin1 polymers per cell (**e**). Data are Mean ± SD. Scale bar, 20 μm. **f** HEK293T cells transfected with the indicated plasmids were subjected to Co-IP. IP and WCL samples were examined by Western blot. HCT116 cells were transfected with siCtrl and siTRIM15 for 72 h, followed by Co-IP using IgG or anti-Axin1 (**g**) or anti-β-TrCP (**h**) antibodies. IP and WCL were analyzed by Western blot. Cells were treated with MG132 (20 μM) for 6 h before lysis. **i** An in vitro kinase assay was performed by incubation of GST-β-catenin with the indicated proteins purified from HEK293T cells. The samples were analyzed by Western blot. GST-β-catenin was purified from bacteria and shown by Coomassie blue staining.

Accumulating evidence shows that both Axin1 stability and polymerization control Wnt signaling activation [25]. We therefore investigated whether TRIM15 could regulate Axin1 polymerization. If TRIM15 had the potential to inhibit Axin1 polymerization, there must be a strong association between Axin1 and TRIM15. We then applied surface plasmon resonance (SPR) to analyze the TRIM15-Axin1 affinity in real time. As shown in Fig. 3a and b, the dissociation constant (*K*_d_) between TRIM15 and Axin1 is moderately stronger than that between Axin1 molecules themselves (37.8 nM versus 147 nM), implying that TRIM15 has the potential to impede the polymerization of Axin1. Notably, the interactions between Axin1 molecules were strengthened when TRIM15 was silenced in HCT116 cells, as revealed by a sucrose gradient centrifugation assay showing that Axin1 was incorporated into larger complexes (Fig. 3c; Extended Data Fig. S3a). This phenomenon was further confirmed in another colon cancer cell line, DLD-1 (Extended Data Fig. S3b). Endogenous Axin1 can form dot-like structures (indicative of Axin1 polymerization) under specific cellular contexts [26]. We therefore detected whether TRIM15 knockdown led to the formation of Axin1 polymerization using immunofluorescence. As expected, TRIM15 silencing obviously exacerbated the formation of Axin1 polymerization (Fig. 3d and e). In contrast, TRIM15 overexpression weakened the association between Axin1 molecules in a dose-dependent manner (Fig. 3f), which was attributed to the fact that Axin1 was predisposed to bind to TRIM15 rather than to itself. In a word, these data strongly indicate that TRIM15 regulates Axin1 polymerization without affecting its stability.

Since Axin1 polymerization is critical for the assembly of the β-catenin destruction complex [16], we therefore investigated whether TRIM15 regulates the complex formation through Axin1. As shown in Fig. 3g, Axin1 had a stronger interaction with GSK3β and phosphorylated β-catenin when TRIM15 was knocked down. Moreover, once TRIM15 was silenced, β-TrCP bound more strongly to β-catenin (Fig. 3h), suggesting that TRIM15 deficiency dramatically induced the formation of the Axin1-mediated destruction complex. To obtain further evidence, we purified proteins from HEK293T cells (Extended Data Fig. S3c) and carried out in vitro kinase assays. Expectedly, Axin1 enhanced GSK3β-phosphorylated β-catenin (Fig. 3i). Importantly, addition of TRIM15 reduced the phosphorylation level of β-catenin (Fig. 3i). Accordingly, ectopic expression of TRIM15 remarkably increased the protein level of nuclear β-catenin, as demonstrated by immunoblotting (Extended Data Fig. S3d) and immunofluorescence (Extended Data Fig. S3e) in HCT116 cells. Notably, Wnt3a, in combination with TRIM15, could also lead to increased nuclear accumulation of β-catenin and greater activation of Wnt signaling (Extended Data Fig. S3f and S3g). Together, TRIM15 is a negative regulator for Axin1 polymerization, the inhibition of which can lead to β-catenin instability and Wnt signaling inactivation.

Next, we conducted molecular dynamics simulation analyses to elucidate the mechanism by which TRIM15 inhibits Axin1 polymerization. As shown in Extended Data Fig. S4a, Axin1-E (the DIX domain of Axin1) is capable of forming a dimer through hydrogen bonding interactions mediated by polar amino acids [22]. Notably, a potential binding was also observed between Axin1-D and Axin1-E, which was similarly facilitated by hydrogen bonding (Extended Data Fig. S4b). This observation suggests that the D domain of Axin1 has the potential to modulate the formation of Axin1 polymers mediated by the E domain. Furthermore, in the absence of TRIM15 binding, the D and E domains of Axin1 exhibited significant conformational flexibility, allowing them to swing freely (Extended Data Fig. S4c). This arbitrary mobility enhanced the formation of dimers or even oligomers between the E domains of different Axin1 molecules. However, due to the presence of TRIM15 binding to the D domain, the swing ability of the E domain was decreased (Extended Data Fig. S4c). Therefore, the binding of TRIM15-Axin1 restricted the free movement of the E domain, albeit not completely. Consequently, the mobility of the E domain was weakened, ultimately impeding the formation of dimers or oligomers between the E domains of different Axin1 molecules. To support this, Axin1 lacking the D domain could still engage in intermolecular binding, whereas TRIM15 lost the ability to disrupt the interaction between Axin1 molecules (Extended Data Fig. S4d). Certainly, more robust evidence relies on structural biology analysis.

### TRIM15 promotes colon cancer cell growth through Wnt/β-catenin signaling

Whether TRIM15 is required for colorectal cancer cell growth? We firstly used two independent *TRIM15* siRNAs to silence endogenous *TRIM15* in two colon cancer cell lines, HCT116 and DLD-1. As shown in Fig. S5a, TRIM15 knockdown decreased the nuclear β-catenin protein levels, with a concomitant reduction of Axin2 and c-Myc. Accordingly, Wnt activation was down-regulated in TRIM15-silenced cells (Extended Data Fig. S5b). In line with this, cell proliferation was obviously inhibited (Extended Data Fig. S5c), and tumor progression was also suppressed following TRIM15 knockdown (Extended Data Fig. S5d and S5e). Notably, the knockdown of TRIM15, mediated by short-hairpin RNA (shRNA), resulted in a reduction of Axin2 but had no effect on Axin1 (Extended Data Fig. S5f). Similarly, cell proliferation and colony formation were also prohibited in TRIM15 knockdown cells (Extended Data Fig. S5g and S5h).

**Fig. 4:**
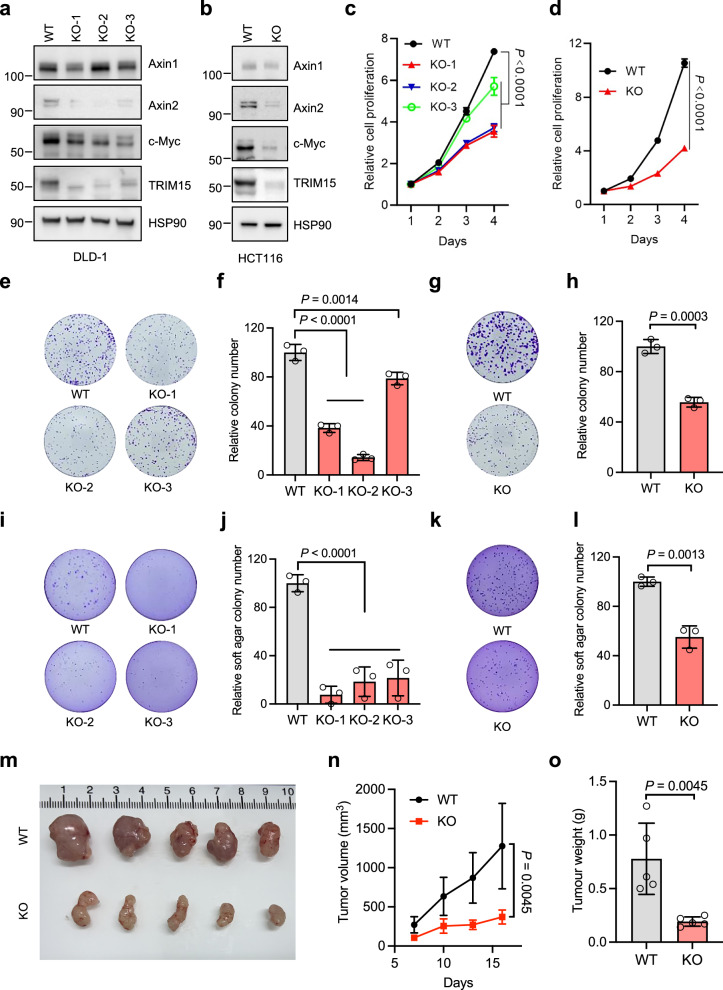
TRIM15 is required for colorectal cancer cell growth. DLD-1 cells stably expressing control or three independent TRIM15 sgRNAs were analyzed. The cells were analyzed using Western blot with the specified antibodies (**a**), and their proliferation was assessed by CCK8 (**c**), colony formation (**e**, **f**), and soft agar colony formation assays (**i**, **j**). HCT116 cells stably expressing control or TRIM15 sgRNA were analyzed. The cells were assessed using Western blot with the specified antibodies (**b**), and their proliferation was evaluated by CCK8 (**d**), colony formation (**g**, **h**), soft agar colony formation (**k**, **l**), as well as tumor formation in xenografted mice, with tumor images (**m**), tumor volume (**n**), and tumor weights shown (**o**) (*n* = 5 biologically independent animals). Data are Mean ± SD. Two-tailed Student’s *t*-test for (**d**, **h**, **l**, **n**, and **o)**. One-way ANOVA for (**c**, **f**, and **j)**.

To further validate the essential role of TRIM15 for colon cell survival, we generated *TRIM15* knockout (*TRIM15*-KO) cells using CRISPR-Cas9 technology. As shown in Fig. 4a, Axin1 expression remained unchanged in three independent *TRIM15*-KO DLD-1 cell lines, consistent with the observation that TRIM15 controlled Axin1 polymerization rather than its stability. Notably, the expression of Axin2 and c-Myc, Wnt target genes, was overtly declined (Fig. 4a). Similar results were obtained in HCT116 cells (Fig. 4b). Consequently, upon TRIM15 knockout, cell proliferation of DLD-1 and HCT116 cells was dramatically inhibited (Fig. 4c and d). Colony formation assays further proved that *TRIM15* KO cells formed smaller and fewer colonies compared with wild type (WT) cells (Fig. 4e–h). Furthermore, TRIM15 KO also impeded the anchorage-independent growth of DLD-1 cells and HCT116 cells (Fig. 4i–l). In xenograft models, tumors derived from TRIM15-KO cells exhibited a significantly slower progression compared to those generated from wild-type cells (Fig. 4m–o).

To establish a functional link between TRIM15 and Wnt/β-catenin signaling in proliferation, we employed a dual rescue strategy. In *TRIM15*-deficient cells, we observed a partial rescue of the proliferation defect upon overexpression of a constitutively active β-catenin S37A mutant (Extended Data Fig. S5i). Conversely, the enhanced proliferation driven by TRIM15 overexpression was effectively blocked by ICG-001, a specific inhibitor of Wnt/β-catenin signaling (Extended Data Fig. S5j). To sum up, these results demonstrate that TRIM15 is critical for the growth of colon cancer cells through modulating Wnt/β-catenin signaling.

### Conditional deletion of *Trim15* in the mouse intestine inhibits tumor formation

**Fig. 5:**
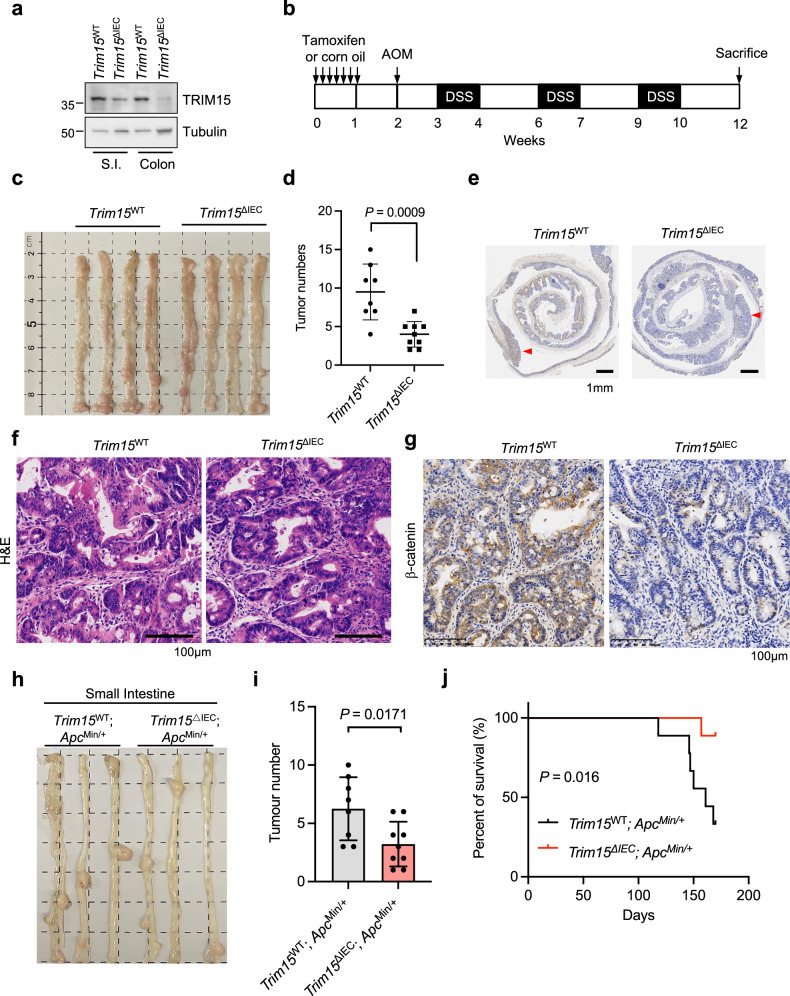
Conditional deletion of *Trim15* in the intestine inhibits tumor formation in CRC mouse models. **a** Immunoblotting detects the protein level of TRIM15 in the small intestine and colon from Tamoxifen-induced *Trim15*^flox/flox^; *Vil-creERT2* mice (designated as *Trim15*^ΔIEC^) compared to *Trim1*5^WT^ mice that received corn oil but not Tamoxifen. S.I. represents the small intestine. **b** Schedule of AOM/DSS-induced CRC mouse model. Representative gross macroscopic image of AOM/DSS-induced tumor formation in colon (**c**), and the polyp number (**d**) from *Trim15*^WT^ mice (*n* = 8) and *Trim15*^ΔIEC^ mice (*n* = 9). **e** Representative images of Swiss-rolled colons from *Trim15*^WT^ mice and *Trim15*^ΔIEC^ mice. The red arrow indicates the location of the tumors. Scale bar, 1 mm. H&E staining of representative tumor sections (**f**) and IHC staining of the protein level of β-catenin in tumors derived from *Trim15*^WT^ and *Trim15*^ΔIEC^ mice (**g**). Scale bar, 100 μm. Representative gross macroscopic image of the small intestines (**h**) and the number of polyps (**i**) were shown for both *Trim15*^WT^; *Apc*^Min/+^ mice (*n* = 8) and *Trim15*^ΔIEC^; *Apc*^Min/+^ mice (*n* = 9). **j** Kaplan–Meier survival curves of *Trim15*^WT^; *Apc*^Min/+^ mice (*n* = 9) and *Trim15*^ΔIEC^; *Apc*^Min/+^ mice (*n* = 9). Data are Mean ± SD. Two-tailed Student’s *t*-test for (**d**, **i**). Log-rank (Mantel–Cox) test for (**j**).

To further explore the biological significance of TRIM15 in regulating Wnt signaling, we generated *Trim15* conditional knockout mice. To achieve specific intestinal deletion of *Trim15*, we crossed *Villin-creERT2* mice with mice carrying the conditional alleles of *Trim15* (*Trim15*^flox/flox^), resulting in *Trim15*^flox/flox^; *Villin-creERT2* mice whose *Trim15* was deleted in intestinal epithelial cells (*Trim15*^ΔIEC^) after tamoxifen treatment (Extended Data Fig. S6a). Genotyping, qPCR, and immunoblotting verified the tamoxifen-administered outcome, respectively (Extended Data Fig. S6b and S6c; Fig. 5a). Azoxymethane (AOM) and Dextran sulfate sodium (DSS) are used to generate a mouse CRC model [27]. Of note, AOM/DSS-induced tumor formation is attributed not only to inflammation-related abnormal proliferation of intestinal epithelial cells [28], but also to the excessive activation of Wnt/β-catenin signaling [29–31]. We therefore induced mouse CRC by using a standard regime [27] (Fig. 5b). As shown in Fig.5c, *Trim15*^ΔIEC^ mice exhibited alleviated tumorigenesis after AOM/DSS genotoxic treatment. The number of tumors in *Trim15*^ΔIEC^ mice was approximately 2-fold lower than that in *Trim15*^WT^ mice (Fig. 5d), suggesting that *Trim15* was required for AOM/DSS-induced tumorigenesis. To further establish a connection between TRIM15 and Wnt/β-catenin signaling in mice, we dissected the tumors derived from *Trim15*^∆IEC^ and *Trim15*^WT^ mice and performed IHC and H&E staining (Fig. 5e and f). As shown in Fig. 5g, genetic ablation of *Trim15* dramatically decreased the expression level of β-catenin induced by AOM/DSS treatment, suggesting that TRIM15 may positively regulate Wnt signaling in vivo. This finding aligns with the notion that TRIM15 contributes to Wnt signaling in colorectal cancer cells.

The *Apc*^Min/+^ mouse is another well-established spontaneous intestinal cancer model that recapitulates key features of human familial adenomatous polyposis (FAP) and colorectal cancer [32]. To investigate the role of TRIM15 in this context, we crossed *Trim15*^flox/flox^; *Villin-creERT2* mice with *Apc*^Min/+^ mice to generate *Trim15*^ΔIEC^; *Apc*^Min/+^ mice (Extended Data Fig. S6d), as confirmed by genotyping and qPCR (Extended Data Fig. S6e and S6f). Consistent with observations in the AOM/DSS colorectal tumor model, *Trim15*^ΔIEC^; *Apc*^Min/+^ mice exhibited a significant reduction in small intestinal tumor burden compared to *Trim15*^WT^; *Apc*^Min/+^ mice (Extended Data Fig. S6g; Fig. 5h and i). In contrast, tumor formation in the colon was rare, with no discernible differences between groups (Extended Data Fig. S6h and S6i). Notably, *Trim15*^ΔIEC^; *Apc*^Min/+^ mice displayed prolonged survival compared to control mice (Fig. 5j). These findings collectively demonstrate that TRIM15 is critical for colorectal tumor progression, likely mediated, at least in part, through its role in activating Wnt/β-catenin signaling.

### *TRIM15* is a target gene of the Wnt/β-catenin signaling pathway

**Fig. 6:**
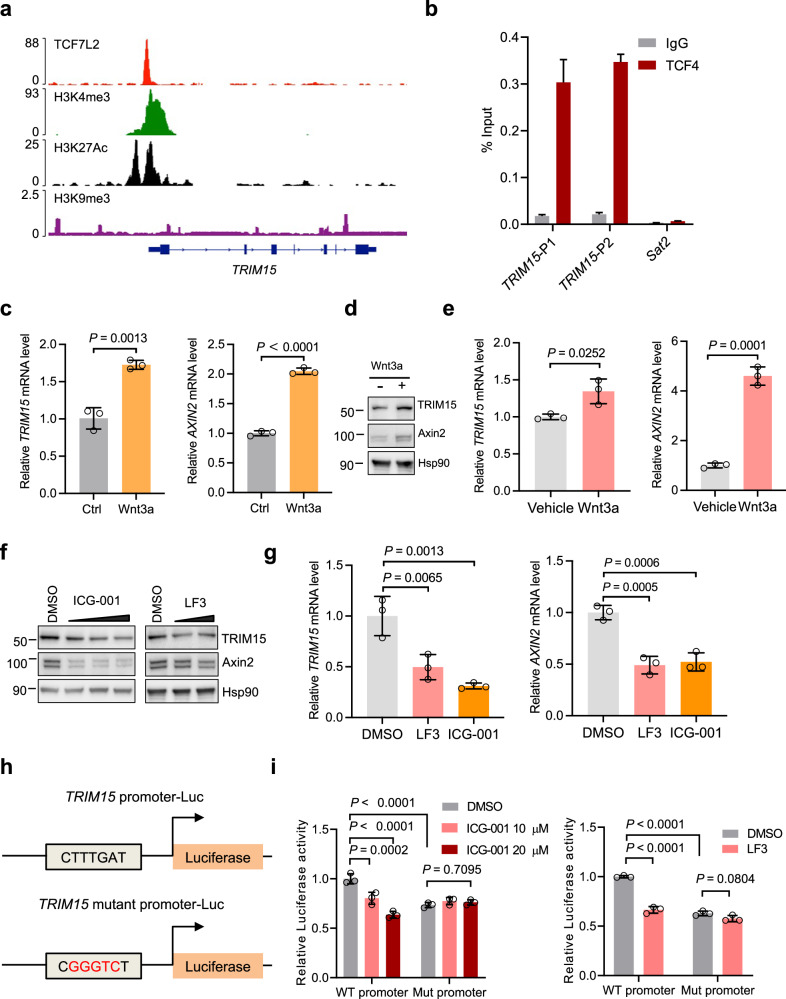
*TRIM15* is a target gene of Wnt/β-catenin signaling. **a** Integrative Genomics Viewer (IGV) analysis of ChIP-seq data in ENCODE of the University of California, Santa Cruz (UCSC), to demonstrate the specific recruitment of TCF7L2, H3K4me3, and H3K27Ac to the *TRIM15* TSS (Transcription start site) in HCT116. **b** Enrichment of the two promoter regions of TRIM15 with anti-TCF4 antibody or IgG as determined by ChIP in HCT116 cells. Results were quantified by qPCR with two *TRIM15* primers (*TRIM15-P1* and *TRIM15-P2*) and a negative *Sat* primer. **c** HEK293T cells were treated with Control medium (Ctrl) or Wnt3a conditioned medium (Wnt3a) for 24 h and then subjected to determine the mRNA level of TRIM15 and Axin2 by qPCR. HCT116 cells were treated with or without Wnt3a (200 μg/mL) for 24 h and then subjected to determine the protein level by Western blot (**d**) and the mRNA level of TRIM15 and Axin2 by qPCR (**e**). **f** HCT116 cells were treated with ICG-001 or LF3 for 24 h at the indicated concentrations and then subjected to determine the protein level of TRIM15 and Axin2 by Western blot. **g** qPCR analysis of the mRNA level of TRIM15 and Axin2 in HCT116 cells treated with ICG-001 (20 μM) or LF3 (20 μM) for 24 h. **h** Schematic diagram of the *TRIM15* promoter-driven luciferase reporter system and its mutant version with site-directed mutations. **i** Dual-Luciferase reporter assay analysis of the activity of *TRIM15* promoter-driven luciferase reporters in response to ICG-001 or LF3 (10 μM) for 24 h. Data are mean ± SD. Two-tailed Student’s *t*-test for (**c**, **e**) One-way ANOVA for (**g**) Two-way ANOVA for (**i**).

TCF7L2/TCF4 is a specific transcriptional factor of Wnt/β-catenin signaling [33]. To our surprise, we observed that TCF7L2, as well as the active epigenetic marks H3K4me3 and H3K27Ac, were all enriched in the promoter of *TRIM15* in HCT116 cells by analyzing the ENCODE database (Fig. 6a). This promoted us to investigate whether *TRIM15* was a direct Wnt target gene. As shown in Fig. 6b, Chromatin Immunoprecipitation (ChIP) assays demonstrated that TCF4 was recruited to the promoter of *TRIM15*, as revealed by qPCR using two different pairs of primers, in HCT116 cells, strongly suggesting that *TRIM15* may be a direct target gene of Wnt signaling in CRC cells. To further confirm this, we firstly used Wnt3a-conditioned medium and Control medium to treat HEK293T cells and found that both *TRIM15* and *AXIN2* were induced (Fig. 6c). Here, *AXIN2*, as the best-known Wnt target gene, is a positive control. Secondly, the commercial Wnt3a protein was applied to treat HCT116 cells, resulting in a remarkable enhancement of both the protein and mRNA levels of TRIM15 and Axin2 (Fig. 6d and e). Conversely, two small molecules, ICG-001 and LF3, which disrupt the interaction between TCF4 and β-catenin [34, 35], were used to treat HCT116 cells, leading to a significant decrease of TRIM15 and Axin2 protein levels in a dose-dependent manner (Fig. 6f). Correspondingly, the mRNA levels of TRIM15 and Axin2 were also reduced (Fig. 6g). These results were further confirmed in DLD-1 cells (Extended Data Fig. S7a and S7b). These findings strongly suggest that Wnt/β-catenin signaling favors TRIM15 expression via transcriptional regulation.

To better characterize *TRIM15* as a Wnt target gene, we identified a potential TCF4 binding site in the promoter sequence of *TRIM15* [36, 37] (Extended Data Fig. S7c). It was so conserved that we constructed it into Luciferase-based reporter plasmids (Fig. 6h). As expected, Wnt3a proteins could activate the luciferase expression, indicating that TRIM15 transcription was enhanced (Extended Data Fig. S7d). Moreover, the mutant plasmid was also generated by site-directed mutations (Fig. 6h). As shown in Fig. 6i, the mutant reporter failed to be promoted, suggesting that endogenous Wnt signaling activated it less effectively than the wild-type reporter. Importantly, ICG-001 and LF3 both efficiently repressed the luciferase expression of the wild-type reporter, but had no such impact on the mutant reporter (Fig. 6i), again confirming *TRIM15* as a Wnt direct target gene. Taken together, these findings suggest that TRIM15 is indeed a Wnt target gene in colon cancer cells, which forms a positive feedback loop that further supports cell proliferation.

### TRIM15 is positively correlated with β-catenin in colorectal tumor patient tissues

**Fig. 7:**
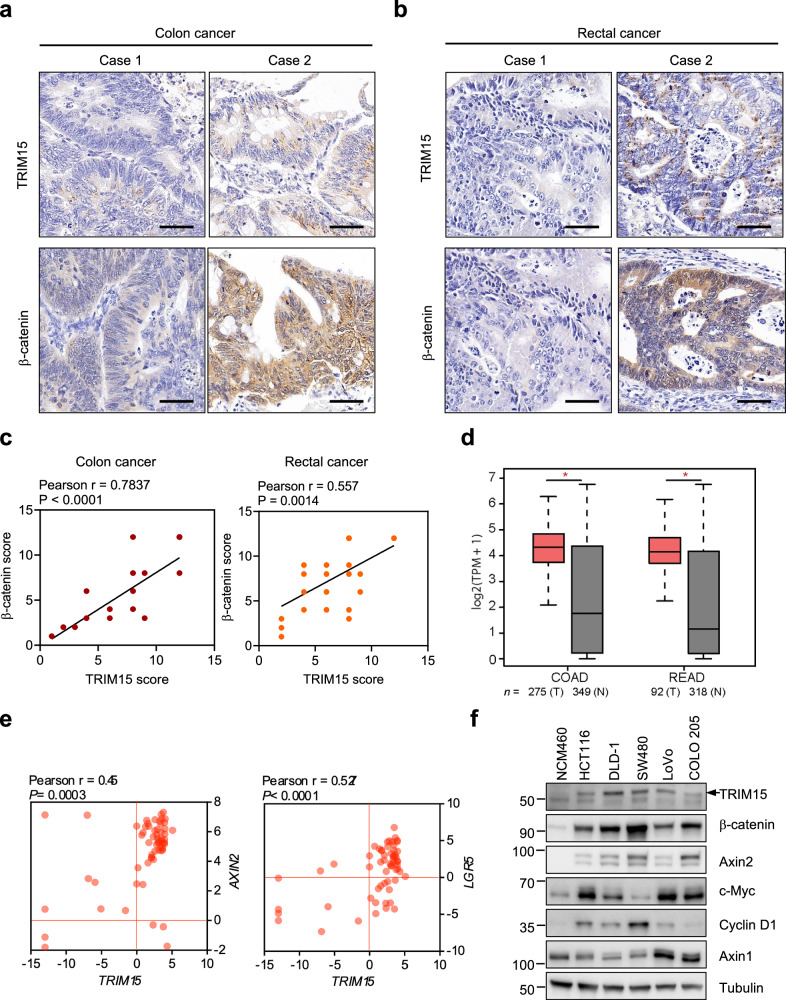
TRIM15 is highly expressed in colorectal cancer and positively correlated with Wnt signaling. IHC analysis of the protein expression levels of TRIM15 and β-catenin in 28 human colon tissues (**a**) and 30 rectal cancer patient tissues (**b**) and statistical analysis of the correlation between the expression levels of TRIM15 and β-catenin (**c**). Scale bar, 50 μm. **d** Analysis of TRIM15 expression in COAD (Colorectal adenocarcinoma) and READ (Rectal adenocarcinoma) compared to normal counterparts by using the GEPIA database (http://gepia2.cancer-pku.cn/). The median levels of TRIM15 were compared between tumors (*n* = 275) and normal tissues (*n* = 349) for COAD, and between tumors (*n* = 92) and normal tissues (*n* = 318) for READ. *P < 0.05. **e** Analysis of the correlation between *TRIM15* and two Wnt target genes *AXIN2* or *LGR5* in colorectal cancer cell lines. The fold change in gene expression (log2 scale) was determined using data from the CCLE. **f** Western blot analysis of total cell lysates derived from NCM460 and five colorectal cancer cell lines with the specified antibodies. One-way ANOVA for (**d**) Pearson correlation for (**c**, **e**).

To determine whether the reciprocal regulation of TRIM15 and Wnt signaling is reflected in human CRC patient tissues, we therefore performed an IHC analysis of human colon and rectal tumor tissues, and discovered that there was a positive correlation between TRIM15 and β-catenin protein levels (Fig. 7a–c). Furthermore, an analysis of public database uncovered that *TRIM15* transcript was higher in colorectal cancer tissues compared with normal tissues (Fig. 7d). In line with this, *TRIM15* was also positively correlated with two Wnt target genes *AXIN2* and *LGR5*, as revealed by analysis of RNA sequencing data of colorectal cancer cell lines from Cancer Cell Line Encyclopedia (CCLE) database (Fig. 7e). When compared with normal human intestinal epithelial cells NCM460, TRIM15 protein levels were found to be specifically overexpressed in human colorectal cancer cell lines (Fig. 7f). Thus, TRIM15, which is highly expressed in colorectal tumors, is correlated with Wnt/β-catenin signaling and promotes colorectal cancer progression. However, analysis of the GEPIA database revealed that the mRNA levels of *TRIM15*, *AXIN1*, *CTNNB1*, and *AXIN2* were not significantly prognostic in CRC (Extended Data Fig. S8a–d).

### The synergistic promotion of Axin1 polymerization and elevated Axin1 protein levels induces cell death

**Fig. 8:**
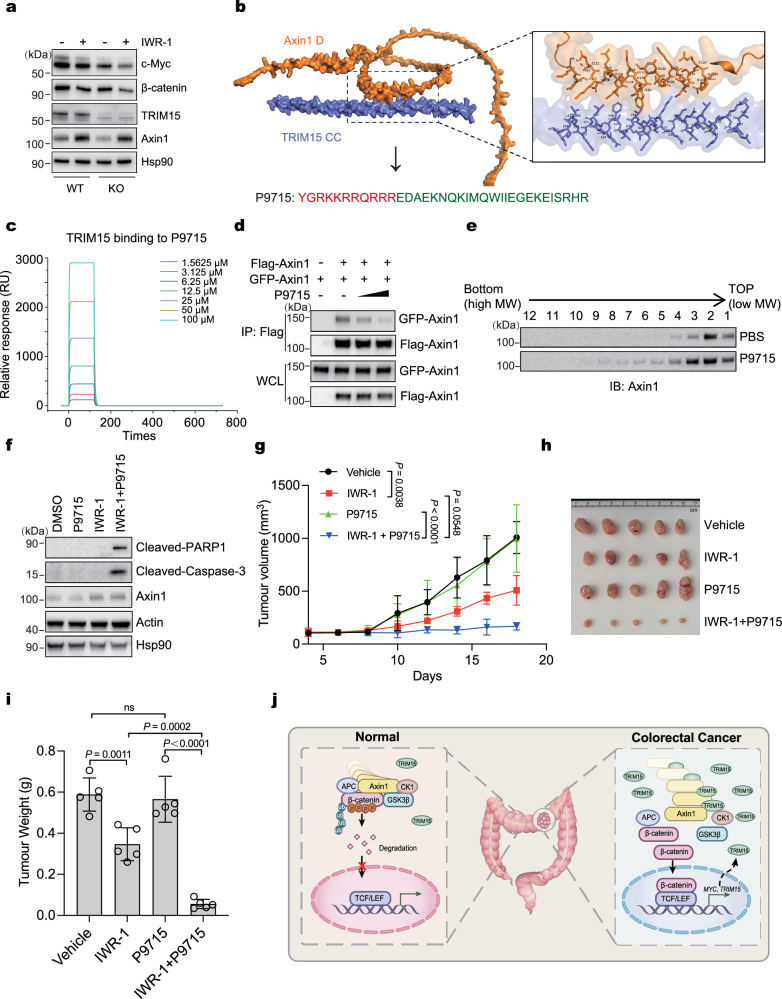
Targeting TRIM15 synergizes with Tankyrase inhibitors to induce cell death. **a** Immunoblotting of total cell lysates from HCT116 wild type and TRIM15 KO cells treated with DMSO or IWR-1 (50 μM) for 48 h. **b** AlphaFold prediction of the complex formed by the Axin1 D domain and TRIM15 CC domain. P9715 included an HIV TAT peptide (red) and a peptide derived from the Axin1 D domain. **c** SPR (BIAcore) analysis of the dissociation constant (*K*_d_) for P9715 from immobilized TRIM15. The interaction between P9715 and TRIM15 had a calculated *K*_d_ of 59.2 μM. **d** HEK293T cells were transfected with the indicated plasmids and then subjected to Co-IP with anti-Flag beads. Cells were treated with P9715 (50 and 100 μM) for 36 h before cell lysis. **e** HCT116 cells were treated with P9715 (100 μM) for 48 h, followed by the sucrose density gradient centrifugation experiment to assess the polymerization degree of Axin1 protein. **f** Immunoblotting of total cell lysates derived from HCT116 cells treated with IWR-1 (50 μM), P9715 (100 μM), or their combination for 72 h. Xenograft tumor formation by HCT116 cells in nude mice that were treated with IWR-1, P9715, or their combination. The tumor volume over time (**g**) tumor images (**h**) and tumor weight (*n* = 5 animals) (**i**) were shown. **j** The proposed model. Data are Mean ± SD. One-way ANOVA for (**g**, **i**).

The protein stability and polymerization of Axin1 are critical for the assembly of the β-catenin destruction complex. Simultaneously increasing Axin1 protein levels and promoting its polymerization could more effectively inhibit Wnt signaling. As shown in Fig. 8a, IWR-1, an inhibitor of Tankyrase, resulted in a greater reduction in c-Myc and β-catenin levels in *TRIM15*-KO cells compared to control cells. Furthermore, we predicted the structural interaction between the TRIM15 Coiled-Coil and the Axin1 D domain using AlphaFold. The docking analysis revealed a potential binding interface between TRIM15 and Axin1 (Fig. 8b). Based on this finding, we designed a cell-penetrating peptide (P9715) derived from the Axin1 D domain to disrupt the Axin1-TRIM15 interaction (Fig. 8b). SPR analysis confirmed that P9715 binds to TRIM15, albeit with moderate affinity (Fig. 8c). Subsequent Co-IP assays demonstrated that P9715 disrupted Axin1 self-association in a dose-dependent manner (Fig. 8d), suggesting that the peptide promotes Axin1 polymerization. Consistent with this, sucrose gradient centrifugation assays showed that P9715 treatment shifted Axin1 toward higher molecular weight fractions (Fig. 8e), indicating enhanced Axin1 polymerization due to weakened TRIM15-Axin1 binding.

Since both Axin1 stabilization and polymerization are required for optimal suppression of Wnt signaling, we combined P9715 and IWR-1 in HCT116 cells to simultaneously elevate Axin1 levels and promote its polymerization. Microscopic examination revealed increased apoptosis in cells treated with both P9715 and IWR-1 (Extended Data Fig. S9a). Immunoblotting further confirmed this observation, as evidenced by the emergence of Cleaved-PARP1 and Cleaved-Caspase-3, two markers of apoptosis (Fig. 8f). Similar pro-apoptotic effects were observed in CT-26 mouse colon cancer cells (Extended Data Fig. S9b), and flow cytometry analysis of Annexin V/PI staining provided additional validation (Extended Data Fig. S9c). To assess the therapeutic potential of this strategy, we employed a xenograft model and found that the combination of P9715 and IWR-1 suppressed tumor growth more effectively than either treatment alone (Fig. 8g-i). These results suggest that dual targeting of TRIM15 (to enhance Axin1 polymerization) and Tankyrase (to increase Axin1 protein levels) represents a promising approach for colon cancer treatment.

Overall, in normal intestinal cells, polymerized Axin1, a crucial scaffold protein, collaborates with other components of the destruction complex to finely regulate the phosphorylation and degradation of β-catenin. In colorectal cancer cells, the overabundance of TRIM15 hampers Axin1 polymerization formation and disrupts the functionality of the degradation complex. Consequently, β-catenin accumulates within the nucleus and triggers downstream oncogene expression. Meanwhile, *TRIM15* as a Wnt target gene in CRC forms a positive feedback, thereby collaboratively promoting CRC progression (Fig. 8j).

## Discussion

Wnt/β-catenin signal pathway plays an important role in CRC initiation and progression, and the involvement of Axin1 in the assembly of the β-catenin destruction complex is pivotal. It has been demonstrated that ERK1/2 activation is dynamically regulated by TRIM15 and CYLD, controlling the growth of both drug-responsive and -resistant melanomas [19]. At the present study, we report that TRIM15, which is highly expressed in colorectal cancer, carries out a non-catalytic function to inhibit Axin1 polymerization, leading to the disassembly of the destruction complex, subsequent β-catenin accumulation, and oncogene transcription. Besides, TRIM15 acts as a Wnt target gene to form a positive feedback loop.

We were amazed to find that TRIM15 expression was the highest in CRC among different types of cancer, implying that it has a critical function in CRC progression. Indeed, TRIM15 knockdown or knockout obviously inhibited CRC cell growth. Furthermore, the favorable role of TRIM15 in CRC formation was proved by using two kinds of mouse colorectal tumor models. Notably, mouse *Trim15* has two short isoforms compared to human *TRIM15*, but these two isoforms still contain the CC domain, which is required for binding to Axin1. Although *Trim15*^ΔIEC^ mice exhibited no obvious phenotype, they developed fewer tumors in both AOM/DSS-induced and *Apc*^Min/+^ CRC models. Importantly, in the AOM/DSS CRC model, the level of β-catenin was significantly reduced in *Trim15*^ΔIEC^ mice, highlighting the role of TRIM15 in promoting tumor formation through the Wnt/β-catenin signaling pathway. Despite its established importance in CRC progression, the mRNA level of *TRIM15* did not serve as a prognostic indicator. We posited that the prognostic potential of TRIM15 might not be fully reflected at the transcriptional level. Post-translational regulation, such as the control of TRIM15 protein stability, could potentially hold greater prognostic value, a hypothesis that warrants further investigation.

Axin1, a central component of the β-catenin destruction complex, has been well-investigated for its protein stability. For example, several E3 ubiquitin ligases (SIAH, RNF46, Smurf2) and Tankyrase negatively regulate the stability of Axin1 via ubiquitination or PARylation, respectively [38–41]. In contrast, with respect to Axin1 polymerization, there has been little investigation, although it is well known that Axin1 polymerization contributes to effective assembly of the destruction complex [16]. It should be noted that the composition of the destruction complex is intricate, and additional components remain untested [42, 43]. We reported that TRIM15 exclusively interacted with Axin1 to inhibit the polymerization of Axin1 independently of its E3 activity. The DIX domain of Axin1 is indispensable for its polymerization [18, 44, 45]. However, our data indicated that the domain adjacent to the DIX domain (D domain of Axin1 in this study) of Axin1 was necessary for interaction with TRIM15. Due to the interaction of certain proteins with the D domain, we cannot exclude the possibility that TRIM15 possibly affects these interactions, thereby influencing the formation of Axin1 polymerization. Notably, the potential for liquid-liquid phase separation in Axin1 does not solely depend on the DIX domain [46, 47], which suggests a sophisticated mechanism of Axin1 polymerization in controlling Wnt signaling. We conducted molecular simulations of the Axin1–TRIM15 complex, which, to some extent, revealed why the binding of TRIM15 to the Axin1 D domain interferes with the formation of Axin1 polymers that are dependent on the DIX domain. However, more investigation is warranted, especially structural biology analysis of the complex.

Small molecules targeting Tankyrase, such as IWR-1 and XAV939, have demonstrated a remarkable inhibitory effect on the proliferation of colorectal cancer cells, as evidenced by previous studies [38, 48, 49]. It is important to highlight that these small molecules were initially developed with the aim of stabilizing Axin1. If specific compounds could be engineered to disrupt the interaction between Axin1 and TRIM15, this would facilitate Axin1 polymerization. Consequently, it would be highly feasible to concurrently augment both the protein level and polymerization of Axin1. This dual-pronged enhancement could more effectively suppress Wnt/β-catenin signaling and, in turn, impede the progression of CRC. Our preliminary data have provided support for this hypothesis. Furthermore, considering that TRIM15 is overexpressed in CRC and functions as a Wnt target gene, targeting TRIM15 may represent a promising therapeutic strategy for CRC driven by aberrant Wnt/β-catenin signaling.

## Materials and methods

### Cell lines

Cells were routinely cultured at 37 °C in a humidified incubator with 5% CO_2_. Cells were purchased from ATCC unless otherwise indicated. NCM460, COLO205, and DLD-1 were cultured in RPMI 1640. HEK293T, SW480, and HeLa were cultured in DMEM. HCT116 was cultured in McCoy’s 5A. LoVo was cultured in F12K. Cell culture medium was made up of 10% fetal bovine serum and 1% penicillin–streptomycin. All cell lines were confirmed negative for mycoplasma contamination.

### Plasmids

HA-TRIM15, Flag-TRIM15, TRIM15-YFP, and Flag-ERK1 were described previously [19]. Flag-Axin1 and GFP-Axin1 were generated by cloning mouse Axin1 cDNA into pcDNA3.1-Flag vectors and pEGFP-C1, respectively. pCDH-Flag-TRIM15 was generated by cloning human TRIM15 into pCDH-EF1-FHC vector. GST-TRIM15, GST-TRIM15 ΔRING (1-61), GST-TRIM15 ΔBBOX (78-119), GST-TRIM15 ΔCC (126-229), GST-TRIM15 ΔRB (1-119), GST-TRIM15 ΔRBC (1-229), and GST-TRIM15 ΔPS (276-465) constructs were generated by cloning the corresponding human cDNA into pGEX-1λT (a modified version of pGEX-4T1). GST-β-catenin and Flag-β-catenin S37A were described previously [50]. pEGFP-Axin1, Axin1-A (1-223), -B (218-436), -C (437-506), -D (507-749), and -E (750-832) were generated [24]. *TRIM15* promoter sequence (-79/40) was inserted into the pGL3-Basic vector, and its corresponding mutant construct was generated by site-directed mutagenesis. These fragments were also cloned into the pcDNA3.1-Flag vector. TRIM15 shRNA and Control shRNA were kindly provided by Walther Mothes. The following plasmids were purchased from Addgene: HA-Ubiquitin WT (#17608), pCDH-EF1-FHC (#64874), and lentiCRISPR V2-Blast (#83480, gift from Mohan Babu).

### Antibodies and other reagents

The following antibodies were purchased from Cell Signaling Technology: Flag (14793), Axin1 (2087), β-TrCP (4394), Phospho-β-Catenin (Ser33/37/Thr41) (9561), HSP90 (4877), c-Myc (5605), Axin2 (2151), non-phospho (Active) β-Catenin (Ser33/37/Thr41) (8814), HA (3724), TCF4/TCF7L2 (2569), HRP-linked rabbit antibody (7074), and HRP-linked mouse IgG antibody (7076). Antibodies against β-Tubulin (TA503129), GFP (TA150052), and HSP90 (TA500494) were purchased from Origene. Antibodies against Lamin A/C (R24823) and GSK3β (340449) were purchased from Zenbio. Antibody against β-Catenin (610154) was purchased from BD Biosciences. Antibody against Axin1 (sc-293190) was purchased from Santa Cruz. Antibody against Axin1 (AF-3287-SP) was purchased from R&D Systems. Antibodies against TRIM15 (13623-1-AP) and Cyclin D1 (26939-1-AP) were obtained from Proteintech. Antibody against TRIM15 (PA5–40946) was purchased from Invitrogen. Anti-Flag beads (A2220) were purchased from Sigma. GFP-Trap magnetic agarose was purchased from Chromotek.

MG132 (S2619), ICG-001 (S2662), and LF3 (S8474) were purchased from Selleck. Wnt3a protein (92276ES10) was purchased from Yeasen. Tamoxifen (T5648) and AOM (A5486) were purchased from Sigma. DSS (0216011080) was purchased from MP Biomedicals.

### Immunoblotting and co-immunoprecipitation assay

For immunoblotting, cells were lysed in lysis buffer (50 mM Tris–Cl at pH 7.4, 150 mM NaCl, 0.5% Triton, 10% Glycerol, supplemented with protease and phosphatase inhibitor cocktail (MCE)) and sonicated at 20% amplitude for 15 s (5 s on, 10 s off) on ice. Then, based on the protein concentration calculated by Bradford protein assays (Vazyme), the samples were resolved by SDS-PAGE and immunoblotting. For Co-IP, cell lysates were immunoprecipitated with the indicated antibodies. The immunoprecipitates were washed four times, with each wash lasting 10 min, using lysis buffer. Afterward, they were subjected to SDS-PAGE and immunoblotting.

### Protein expression and purification

To generate GST and GST-fused proteins from bacteria, E. coli BL21 (DE3) cells, containing the indicated plasmids, were incubated on a shaker at 37 °C for 6 h and then induced to express protein by supplementing with 0.5 mM IPTG. E. coli cells were lysed with lysis buffer (50 mM Tris-HCl at pH 7.4, 0.5% Triton, 200 mM NaCl, 10% glycerol, 1 mM DTT, and 1 mM PMSF). After sonication and centrifugation, lysates were incubated with an appropriate amount of Glutathione Sepharose 4B (GE Healthcare) at 4 °C overnight. The resin was washed three times with lysis buffer containing 500 mM NaCl, followed by two more washes with lysis buffer. The proteins immobilized on Glutathione Sepharose beads were examined using SDS-PAGE and stored at −80 °C. His-TRIM15 proteins from bacteria were obtained through refolding from inclusion bodies. To purify Flag-fused proteins or GFP-fused proteins from mammalian cells, HEK293T cells were transfected with the indicated plasmids, and the proteins were purified using anti-Flag (M2) beads or anti-GFP magnetic beads. The beads were washed three times with lysis buffer containing 800 mM NaCl, followed by two more washes with lysis buffer. The proteins immobilized on anti-Flag or anti-GFP beads were confirmed using SDS-PAGE. Where noted, Flag-fused proteins were eluted using Flag peptides. Specifically, for the SPR experiment, the purified proteins were further concentrated using centrifugal filters (Millipore, UFC5030).

### In vivo ubiquitination assay

The denatured immunoprecipitation (d-IP) assay was used to detect protein ubiquitination in cells. HEK293T cells were lysed in SDS-denaturing buffer (62.5 mM Tris–HCl, pH 6.8, 2% SDS, 10% glycerol, 100 mM DTT) and boiled for 15 min. Cell lysates were then diluted 10-fold in native lysis buffer (50 mM Tris–HCl, pH 7.4, 0.5% Triton, 10 mM NaCl, 10% glycerol). After brief centrifugation at high speed, the supernatants were immunoprecipitated with anti-Flag beads at 4 °C overnight. Finally, the immunocomplexes were washed three times with native lysis buffer, separated by SDS–PAGE, and immunoblotted with the appropriate antibody.

### GST-pulldown assay

Flag-fused proteins were purified from HEK293T cells and incubated with Glutathione Sepharose 4B beads (GE Healthcare), which were bound to either bacterially expressed GST or GST fused proteins, in binding buffer (50 mM Tris–HCl at pH 7.4, 150 mM NaCl, 0.5% Triton, 10% glycerol, 1 mM DTT, protease inhibitor cocktail) at 4 °C for 4 h or overnight. In some cases, Flag-fused proteins were incubated with GFP-Axin1 immobilized on anti-GFP magnetic beads. The beads were washed four times with binding buffer. Both the pull-down samples and the input were examined by Western blot.

### Quantitative real-time PCR analysis (qPCR)

TRIzol^TM^ reagent (15596026CN, Thermo Fisher Scientific) was used to extract total RNA from cells and tissue samples. Subsequently, 1 µg of extracted RNA was reverse transcribed using RevertAid First Strand cDNA synthesis kit (K1622, Thermo Fisher Scientific). The cDNA obtained was amplified using SYBR Green Premix (A57156, Thermo Fisher Scientific), and qPCR reaction was performed on a LightCycler96 (Roche). All primer sequences are as follows: Human *TRIM15*, sense 5’-TCCCTGAAGGTGGTCCATGAG-3’, antisense 5’-CAGGATCTTGCCCGAGGATT-3’; Mouse *Trim15*, sense 5’-TCGTCTCAGGAGTCGGTTAGA-3’, antisense 5’-GCTTTTTGCTTTCAACCTGCATC-3’; Human *AXIN2*, sense 5’-GTGAGGTCCACGGAAACTGT-3’, antisense 5’-TGGCTGGTGCAAAGACATAG-3’.

### Transient and stable RNAi knockdown

For transient knockdown, cells were transfected with siRNAs (Tsingke) using jetPRIME® Transfection reagent (Polyplus) according to the manufacturer’s instructions. The siRNA sequences were as follows: siTRIM15#1: 5’-CCCUGAAGGUGGUCCAUGA-3’; siTRIM15#2: 5’-GCGAGAACGAUGCCGAGUU-3’ [51]; negative control: 5’-UUCUCCGAACGUGUCACGU-3’. For stable knockdown, cells were infected with lentiviral particles expressing Control shRNA or TRIM15 shRNA and subsequently selected with puromycin (1 μg/mL). The specific sequences of shRNAs were: shControl: 5’-TACAAACGCTCTCATCGACAAG-3’, shTRIM15: 5’-AGATGAGATTGAGGATGTA-3’.

### Generation of *TRIM15*-knockout cells

To generate *TRIM15* knockout HCT116 and DLD-1 cell lines, a single guide RNA (sgRNA) targeting *TRIM15* was cloned using DNA oligos and ligated into lentiCRISPR V2-Blast. sgTRIM15 target sequences were: 1, 5’-GCAGAGCAGGATCTTGCCCG-3’; 2, 5’-GATGCGGTGACCATTCCCTG-3’; 3, 5’-CCTGTGGACACACCTTCTGC-3’. HCT116 cells were infected with sgTRIM15-1 lentiviral particles, while DLD-1 cells were infected with either sgTRIM15-2 or sgTRIM15-3 lentiviral particles. The infected cells were then selected with blasticidin at a concentration of 10 μg/mL, and single clones were isolated from the selected cells.

### Cell viability and colony formation assays

Cell proliferation and viability were examined by CCK8 assay, while colony formation was assessed using a crystal violet assay. Forthe CCK8 assay, cells (1–3 × 10^3^ cells/well) were seeded in 96-well plates. Before detection, 10 μL CCK8 was added into each well and incubated at 37 °C for 2 h. The absorbance was then measured at 450 nm. For the crystal violet assay, cells (500–1000 cells/well) were seeded in six-well plates. The medium was changed once every two days until the colonies formed. The cells were stained with crystal violet (0.1% in 20% methanol) at room temperature for 30 min. The results were graphically presented using ImageJ Software and GraphPad Prism 9 (GraphPad Software).

### Soft agar assay

HCT116 and DLD-1 cells (500–1000 cells/well) were mixed with the corresponding medium containing 0.3% agar and plated on top of base agar (0.6%) that had been pre-plated in 6-well plates. After approximately two weeks, colonies were clearly formed and stained with crystal violet. The experiments were performed in triplicate. The number of colonies was calculated using ImageJ and statistically analyzed by GraphPad Prism 9.

### Sucrose gradient centrifugation assay

HCT116 or DLD-1 cells were transfected with Control siRNA or TRIM15 siRNA for 72 h. The cells were collected in pre-cold PBS, centrifuged, and lysed in the lysis buffer (30 mM Tris–HCl, pH 7.5, 150 mM NaCl, 1% Triton X-100, protease inhibitor cocktail and phosphatase cocktail inhibitor) for 20 min on ice. After centrifugation of the lysates at 14,000 rpm for 15 min, the supernatant was layered on top of a sucrose gradient ranging from 10% to 50% (with increments of 10%) in buffer containing 30 mM Tris–HCl, pH 7.5, 150 mM NaCl, 0.02% Triton X-100, protease inhibitor cocktail, and phosphatase cocktail inhibitor. Ultracentrifugation was performed in a Beckman SW41 Ti rotor at 40,000 rpm and 4 °C for 4 h. Fractions were collected and analyzed by Western blot.

### Dual-luciferase reporter assay

Cells were plated in 96-well plates. For the TRIM15 promoter-driven luciferase reporter assay, 140 ng of the reporter plasmid and 10 ng of Renilla were used in each well. For the Wnt reporter assay, 15 ng of Topflash and 0.5 ng of Renilla were used in each well. Other plasmids and empty vectors were co-transfected to ensure the total amount of plasmid DNA in each well was 150 ng. After 24 h, Wnt3a or small molecules were added to the indicated wells for an additional 24 h. Then, the cells were lysed in lysis buffer, and luciferase activity was detected using the Dual-Luciferase Reporter Kit (11402ES60, Yeasen).

### Animal experiments

For the tumor xenograft model, 5-week-old male athymic nude mice (GemPharmatech, Nanjing, China) were used. HCT116 cells transfected with siRNAs or expressing sgCtrl or sgTRIM15 were harvested and suspended in 100 μL 1:1 PBS and Matrigel (Corning). Cells (1 × 10^6^) were injected subcutaneously in the right flank of each mouse. The tumor size was measured using a digital caliper every three days and calculated as *V* = length × width 2/2. Finally, mice were sacrificed, and the tumors were removed and weighed. Notably, nude mice were injected intraperitoneally with P9715 (5 mg/kg), IWR-1 (5 mg/kg), or both every other day when tumor volume reached about 100 mm^3^. For the AOM/DSS CRC model, *Trim15*^flox/flox^ mice (Cyagen Biosciences, Suzhou, China) were crossed with *Vil-creERT2* mice. Finally, 5-week-old *Trim15*^flox/flox^; *Vil-creERT2* mice were used. Tamoxifen or corn oil was injected for 7 days to obtain *Trim15*^ΔIEC^ mice and Control mice. After one week, mice were injected intraperitoneally with 10 mg/mL AOM, followed by normal drinking water for one week. Then, water containing DSS (2.5%) was given for one week, and replaced with normal drinking water for two weeks. This is one cycle. After three consecutive cycles, the experiment was ended, and the mice were sacrificed to gain colonic and rectal tissues. Tissues were fixed with 4% PFA for IHC and H&E staining. For the *Apc*^Min/+^ CRC model (GemPharmatech, Nanjing, China), 5-week-old *Apc*^Min/+^; *Trim15*^flox/flox^; *Vil-creERT2* mice were used. Tamoxifen or corn oil was also injected for 7 days to obtain *Trim15*^ΔIEC^; *Apc*^Min/+^ mice and *Trim15*^WT^; *Apc*^Min/+^ mice. After 12 weeks, mice were sacrificed, and the tumors in the small intestine were analyzed.

### Immunofluorescence

Cells on the slide were fixed with 4% paraformaldehyde for 20 min, permeabilized with 0.2% Triton X-100 in PBS for 10 min, and then blocked with 2% BSA in PBS for 30 min at room temperature. Subsequently, the slides were incubated with the primary antibody against anti-Flag (Cell Signaling Technology, 1:800) or anti-Axin1 (Santa Cruz, 1:100) overnight at 4 °C. The next day, the slides were further incubated with fluorescence-labeled secondary antibodies (Invitrogen, 1:500) for 1 h at room temperature. After washing with PBS, coverslips were sealed with Prolong Diamond Antifade Mountant with DAPI (Thermo Fisher). Images were captured by a Zeiss LSM 910 confocal microscope.

### In vitro kinase assay

GST-β-catenin purified from bacteria was used as the substrate. Flag-Axin1, Flag-TRIM15, and Flag-GSK3β were purified from HEK293T cells. The in vitro kinase assay was performed in the kinase buffer (50 mM Tris–HCl, pH 7.4, 50 mM NaCl, 10 mM MgCl_2_, 1 mM ATP, and 1 mM DTT) at 30 °C for 30 min with gentle agitation. The reactions were stopped by boiling with 2 × SDS loading buffer for 10 min. The samples were then analyzed using SDS–PAGE and immunoblotting with a phospho-β-catenin S33/37/T41 antibody. The presence of GST-β-catenin was confirmed by Coomassie blue staining.

### Immunohistochemistry

Tumor tissue microarrays containing 28 pairs of colon cancer and 30 cases of rectal cancer tissue (Shanghai OUTDO Biotech) were used for IHC. The tissue microarray was baked at 60 °C for 2 h, dewaxed with Xylenes three times for 10 min each, and then rehydrated through an ethanol gradient. Antigen retrieval was performed by heating the slides in a citrate buffer (10 mM sodium citrate) in a microwave for 15 min, followed by natural cooling. The tissue slides were then stained using an IHC kit (Tuling, I20012C). The blocked sections were incubated with antibodies against TRIM15 (Invitrogen, 1:100) and β-catenin (BD Biosciences, 1:200) overnight at 4 °C. The next day, the sections were then incubated with a secondary antibody and stained with DAB. Hematoxylin and Eosin (H&E) staining was performed, followed by dehydration, clearing in xylene, and mounting onto the slide using mounting medium. The slides were then sealed with natural gum. Tissues were visualized and captured using an inverted microscope. The extent of stained cells was indicated as follows: 0 (0%), 1 (1–25%), 2 (26–50%), 3 (51–75%), and 4 (76–100%). The final IHC staining scores were analyzed using Aipathwell software.

### Molecular dynamics simulation

A comprehensive full-sequence blast search was conducted to identify crystal structures within the RCSB PDB database. This search specifically pinpointed the sequence spanning residues 294–465 of TRIM15 (PDB ID: 7QS2) as the relevant segment. Subsequently, one crystal structure, namely PDB ID: 4CG4 [52], was selected as the template for homology modeling to reconstruct the full-length three-dimensional structure of TRIM15. Conversely, in the case of Axin1, no suitable homology modeling template was identified. Therefore, the predicted structure for Axin1 was directly obtained from the AlphaFold database (entry AF-O35625-F1). Water molecules, co-crystallized ligand, and ions in protein TRIM15 were removed for further docking process. ZDOCK software was used for the protein-protein docking predictions as described in the literature [53]. Molecular dynamics (MD) simulation was used to study the conformational change and protein-protein interaction between Axin1 and TRIM15 using the methods previously described [54–57].

### Surface plasmon resonance (SPR)

SPR experiment was performed using a BIAcore 8 K instrument (Cytiva, USA). To analyze the *K*_d_ of Axin1 or TRIM15 binding to Axin1, a CM5 series S sensor chip (Cytiva, USA) was activated with 0.1 M N-Hydroxysuccinimide (NHS; Sangon, A600526) and 0.4 M 1-(3-dimethylaminopropyl)-3-ethylcarbodiimide hydrochloride (EDC; Sangon, C600433). Flag-Axin1 protein was immobilized on the activated chip surface by amine coupling, using a working concentration of 20 μg/mL in sodium acetate buffer (pH 4.5). Flow cell 1 was left empty and used as a reference flow cell for background subtraction. Flow cell 1 and flow cell 2 were then blocked with 1 M ethanolamine hydrochloride solution (pH 8.5) (Shanghai Yuanye Bio-Technology, R28421). The analytes were injected at a flow rate of 30 μL/min, with an association time of 120 s and a dissociation time of 300 s. The surface was regenerated by injecting glycine-HCl (pH 2.5) at the same flow rate, generating a series of sensorgrams. The analyte concentration range was 25–400 nM (2-fold dilution series). To analyze the *K*_d_ of TRIM15 binding to P9715 (synthesized by Hangzhou Go Top Peptide Biotech Co., Ltd), His-TRIM15 protein was used at 40 μg/mL in sodium acetate buffer (pH 4.0). The analyte peptide, P9715, was kept in DPBS buffer. The analyte was injected at a flow rate of 30 μL/min, with an association time of 120 s and a dissociation time of 600 s. Data analysis was performed using BIAcore Insight Epitope Binning Extension software.

### Statistics

For animal studies, the sample size was chosen based on our laboratory’s extensive experience. Animals were randomly assigned to either the treatment or control group. Only those that survived the entire duration of the experiment and showed no signs of unrelated severe illness were included in the final analysis. Statistical analyses were performed using Graphpad Prism 9 or Microsoft Excel 2016. A two-tailed Student’s *t*-test was used to evaluate differences between two groups. One- or two-way ANOVA was used to evaluate the statistical significance among multiple groups. A *P* < 0.05 was considered to be statistically significant.

### Ethics approval and Consent to participate

The tumor tissue microarrays used in this study were commercially purchased from Shanghai Outdo Biotech Company (Shanghai, China). All human tissue samples were collected with appropriate ethical approval and informed consent from donors, in compliance with relevant ethical guidelines and regulations. This study involving human participants was reviewed and approved by the Shanghai Outdo Biotech Company’s Ethics Committee (Approval No. SHXC2021YF01). All animal experiments were performed according to relevant guidelines and regulations and were approved by the Ethics Committee of the Fourth Affiliated Hospital, Zhejiang University School of Medicine (ZJU20230330).

## Supplementary information

Supplementary figure legends

Extended Data Figure 1

Extended Data Figure 2

Extended Data Figure 3

Extended Data Figure 4

Extended Data Figure 5

Extended Data Figure 6

Extended Data Figure 7

Extended Data Figure 8

Extended Data Figure 9

## Data Availability

All data that support the findings of this study are available from the corresponding authors upon reasonable request.
