# Supplementary material for: Targeting TRIM15-mediated Axin1 depolymerization suppresses Wnt signaling and inhibits colorectal cancer growth
Source: Cell Death Dis. 2025 Dec 29;17(1):152. doi: 10.1038/s41419-025-08400-7 (PMC12859064; doi:10.1038/s41419-025-08400-7)
Supplement: Supplementary file 1 — Supplementary figure legends [file 41419_2025_8400_MOESM1_ESM.docx]

**Extended Data Fig. 1 TRIM15 is overexpressed in CRC and interacts with Axin1**

**a-c,** Analysis of the expression levels of TRIM15 in the NCI-60 tumor cell lines **(a)**, GEPIA database **(b)** and HPA database **(c)**.

**d,** HEK293T cells were transfected with GFP-Axin1 along with or without Flag-TRIM15 and then subjected to Co-IP using anti-Flag beads. IP samples and WCL were analyzed by Western blot.

**e,** HEK293T cells were transfected with GFP-Axin1 or GFP-Axin1 ΔDIX along with or without Flag-TRIM15 and then subjected to Co-IP using anti-Flag beads. IP samples and WCL were analyzed by Western blot.

**Extended Data Fig. 2 TRIM15 Coiled coil domain is necessary for association with Axin1**

**a,** Schematic illustration of the full-length TRIM15 and its truncated mutations. R, RING; B, BBOX; CC, Coiled coil; PS, PRY and SPRY.

**b,** HEK293T cells were transfected with GFP-Axin1 along with TRIM15 or truncated TRIM15 mutations and then subjected to Co-IP. IP samples and WCL were analyzed by Western blot.

**Extended Data Fig. 3 TRIM15 contributes to Wnt/β-catenin signaling**
**a,** Immunoblotting of TRIM15 in siCtrl or siTRIM15 transfected HCT116 cells for 72 h.

**b,** DLD-1 cells were transfected with siCtrl and siTRIM15. Following a 72-hour incubation, the sucrose density gradient centrifugation assay was performed to assess the extent of Axin1 polymers.

**c,** Flag-Axin1, Flag-GSK3β, and Flag-TRIM15 purified from HEK293T cells were shown by SDS-PAGE and Coomassie blue staining. Arrow indicates these proteins.

**d, e,** HCT116 cells were transfected with Empty vector (EV) or TRIM15-YFP and subjected to Western blot **(d)** and immunofluorescence staining **(e)**. Scale bar, 20 μm.

**f,** Cytosolic and nuclear fractions of HEK293T cells, which were transfected with EV or TRIM15-YFP and then treated with or without Wnt3a medium for 24 h, were analyzed by Western blot using specified antibodies.

**g,** The promotion of Wnt3a-induced Wnt activity by TRIM15 was assessed using the TOPflash reporter assay.

**Extended Data Fig. 4 Molecular simulation of the TRIM15-Axin1 complex**

**a,** Simulated protein complexes of DIX domains of Axin1 proteins (denoted as Axin1-E for clarity in this study) forming stable dimers with favorable interactions. Green and bright brown colors represent the two molecules of Axin1-E, respectively.

**b,** Simulated protein complexes of Axin1-D and Axin1-E showed interactions between them. Blue and bright brown colors represent Axin1-D and Axin1-E, respectively.

**c,** The morphology of the D and E regions of Axin1 protein without TRIM15 interaction (Above). The morphology of the D and E regions of Axin1 protein following its interaction with TRIM15 protein (Below).

**d,** HEK293T cells were transfected with the indicated plasmid and then subjected to Co-IP. IP and WCL samples were examined by Western blot.

**Extended Data Fig. 5 TRIM15 is required for colorectal cancer cell proliferation**

**a, c,** DLD-1 and HCT116 were transfected with either siCtrl or siTRIM15s. WCL and nuclear samples were analyzed by Western blot **(a)**, and cell proliferation was tested using CCK8 assay **(c)**.

**b,** TOPflash reporter assay analysis of Wnt activity in HCT116 cells transfected with either siCtrl or siTRIM15s.

**d, e,** HCT116 cells transfected with siCtrl or siTRIM15 were subcutaneously injected into nude mice. After two weeks, the mice were euthanized, and the tumors were excised, photographed (**d**), and weighed **(e)**.

**f-h,** DLD-1 cells stably expressing Control shRNA (shCtrl) or TRIM15 shRNA (shTRIM15) were examined by Western blot **(f)**, proliferation was assessed by CCK8 assay **(g)**, and the relative colony number was analyzed **(h)**.

**i,** Proliferation of HCT116 WT and *TRIM15* KO cells with or without β-catenin S37A was assessed by CCK8 assay. Data were shown as the ratio of absorbance on day 4 to that on day 1.

**j,** HCT116 cells stably expressing TRIM15 were examined by Western blot (left) and for proliferation (right). For the proliferation assay, cells were treated with or without 10 μM ICG-001 for 24 h was assessed by CCK8 (right). Data were shown as the ratio of absorbance on day 2 to that on day 1.

Data are Mean ± SD. Two-tailed Student’s t-test for **c**, **e**, **g, h**; One-way ANOVA for **b, i, j**.

**Extended Data Fig. 6 Two CRC mouse models**

**a, b,** A breeding schematic for mice of two genotypes, *TRIM15*^flox/flox^; Vil-CreERT2 (*Trim15*^WT^) and tamoxifen-induced *Trim15*^flox/flox^; Vil-CreERT2 (*Trim15*^ΔIEC^) **(a)**. The genotyping was examined by agarose gel electrophoresis **(b)**.

**c,** qPCR analysis of the mRNA level of Trim15 in *Trim15*^WT^ mice and *Trim15*^ΔIEC^ mice.

**d, e,** A breeding schematic for mice of two genotypes, *Trim15*^flox/flox^; *Apc*^Min/+^; Vil-CreERT2 (*Trim15*^WT^; *Apc*^Min/+^) and tamoxifen-induced *Trim15*^flox/flox^; *Apc*^Min/+^; Vil-CreERT2 (*Trim15*^ΔIEC^; *Apc*^Min/+^) **(d)**. The genotyping was examined by agarose gel electrophoresis **(e)**.

**f,** Schematic illustration of conditional deletion of *Trim15* in *Trim15*^flox/flox^; *Apc*^Min/+^; *Vil-cre ERT2* mice.

**g,** qPCR analysis of the mRNA level of Trim15 in *Trim15*^WT^; *Apc*^Min/+^ mice and *Trim15*^ΔIEC^; *Apc*^Min/+^ mice.

**h, i,** Representative gross macroscopic image of the colons (**h**) and the number of polyps **(i)** were shown for both *Trim15*^WT^; *Apc*^Min/+^ mice (n = 8) and *Trim15*^ΔIEC^; *Apc*^Min/+^ mice (n = 9). Data are Mean ± SD. Two-tailed Student’s *t*-test for **c**, **g**, **i**.

**Extended Data Fig. 7 Wnt/β-catenin signaling controls TRIM15 transcription**

**a,** Immunoblotting of TRIM15 in DLD-1 cells treated with ICG-001 at the indicated concentrations for 24 h.

**b,** qPCR analysis of the mRNA level of TRIM15 in DLD-1 cells treated with ICG-001 (20 μM) for 24 h.

**c,** The DNA sequence of human *TRIM15* promoter region (-499 to 100). The red indicates TCF4-conserved binding motif in *TRIM15* promoter sequence.

**d,** Dual-luciferase reporter assay analysis of the activity of *TRIM15* promoter-driven reporter-induced by Wnt3a protein (200 μg /mL) for 24 h in HCT116 cells.

**Extended Data Fig. 8 Kaplan-Meier survival analyses**

**a**-**d**, Kaplan-Meier curves of colorectal cancer patients with high and low expression of *TRIM15* mRNA, *AXIN1* mRNA, *CTNNB1* mRNA and *AXIN2* mRNA in the GEPIA database.

**Extended Data** **Fig. 9 P9715 synergizes with IWR-1 to induce cell apoptosis**

**a,** Representative images showing that the induction of apoptosis in HCT116 cells treated with IWR-1 (50 μM), P9715 (100 μM), or their combination for 72 h.

**b,** Immunoblotting of total cell lysates derived from CT26 mouse colon cancer cells treated with IWR-1 (50 μM), P9715 (100 μM), or their combination for 72 h.

**c,** FACS analysis of HCT116 cells stained with Annexin V-PE and PI following 72 h treatment with IWR-1 (50 μM), P9715 (100 μM), or their combination.
