# Supplementary figures and images for: Targeting TRIM15-mediated Axin1 depolymerization suppresses Wnt signaling and inhibits colorectal cancer growth
Source: Cell Death Dis. 2025 Dec 29;17(1):152. doi: 10.1038/s41419-025-08400-7 (PMC12859064; doi:10.1038/s41419-025-08400-7)

**a**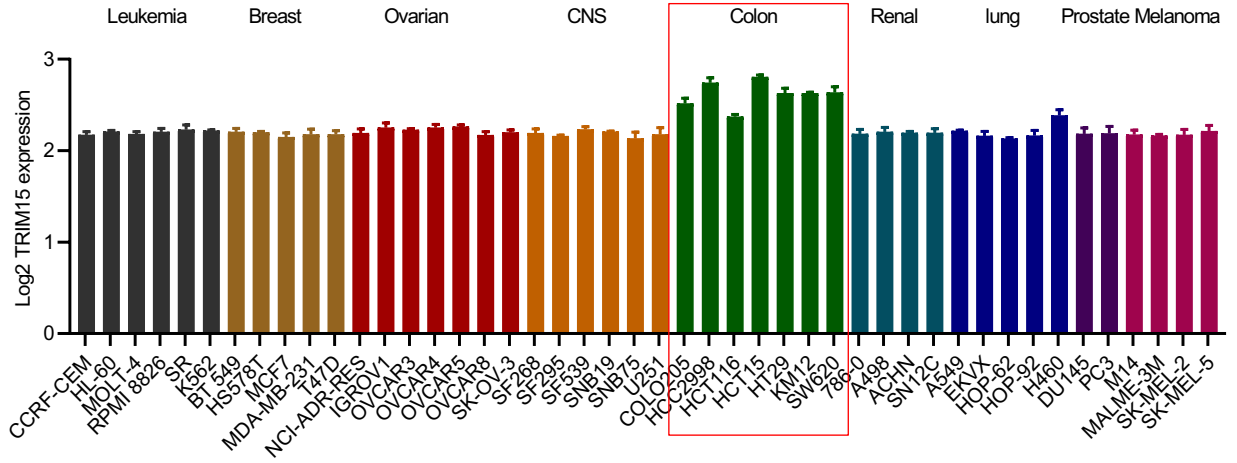**b**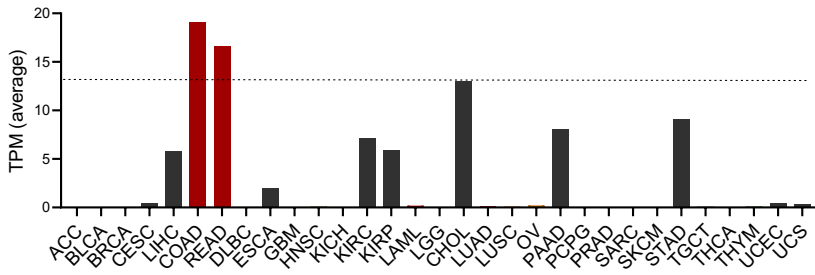**d**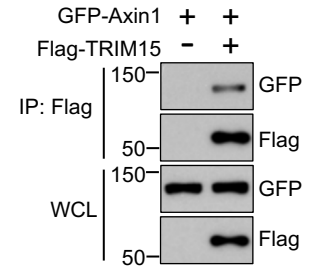**c**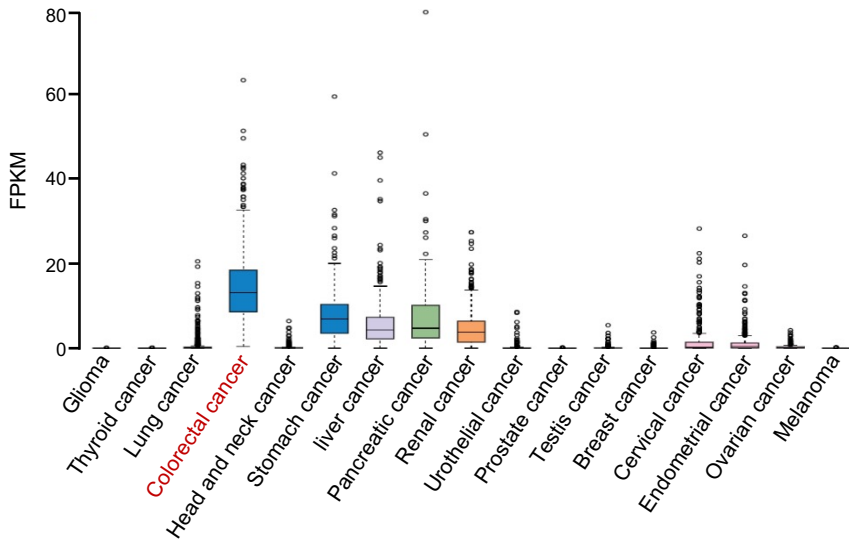**e**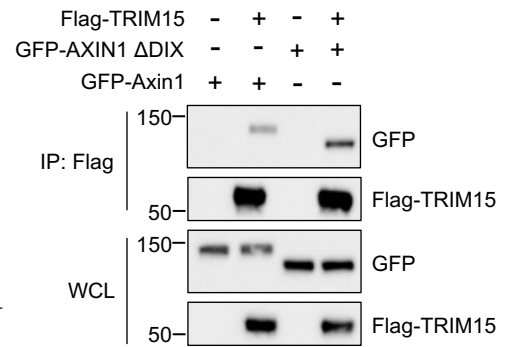

Supplement: Supplementary file 2 — Extended Data Figure 1 [file 41419_2025_8400_MOESM2_ESM.pdf]

**a**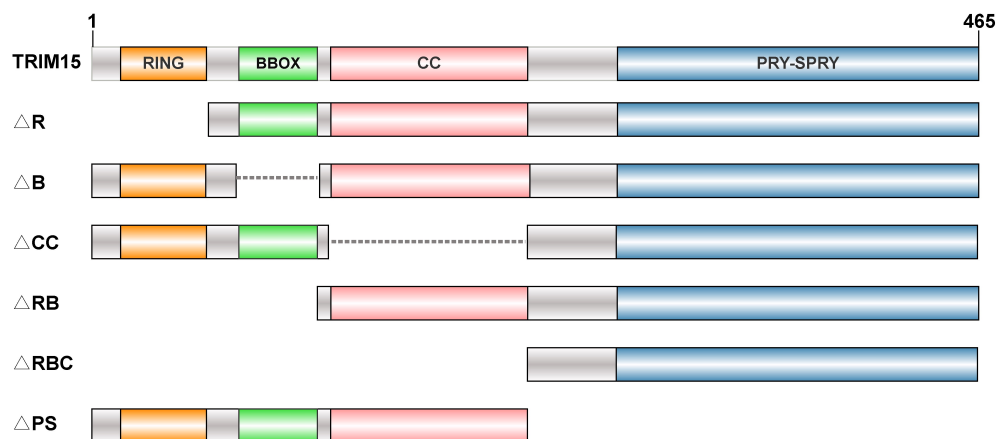**b**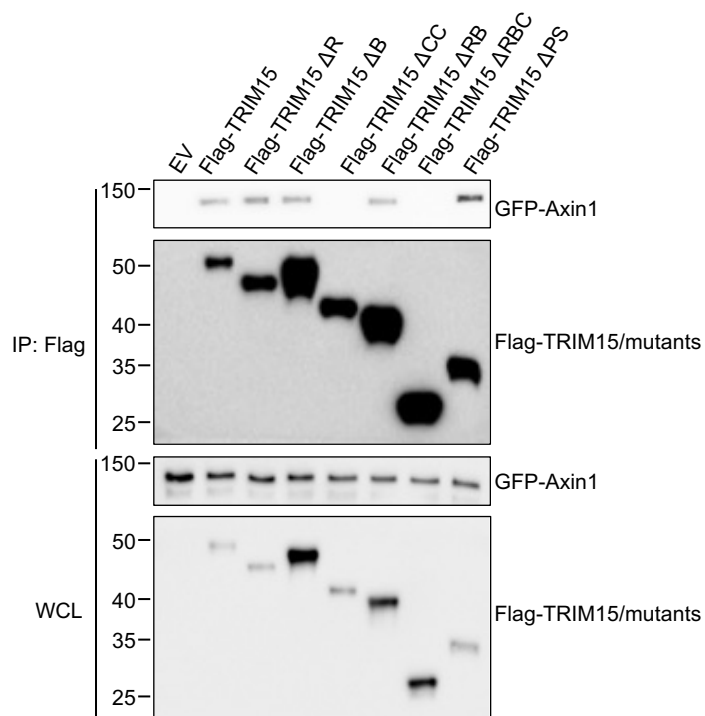

Supplement: Supplementary file 3 — Extended Data Figure 2 [file 41419_2025_8400_MOESM3_ESM.pdf]

**a**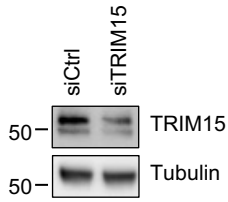**b**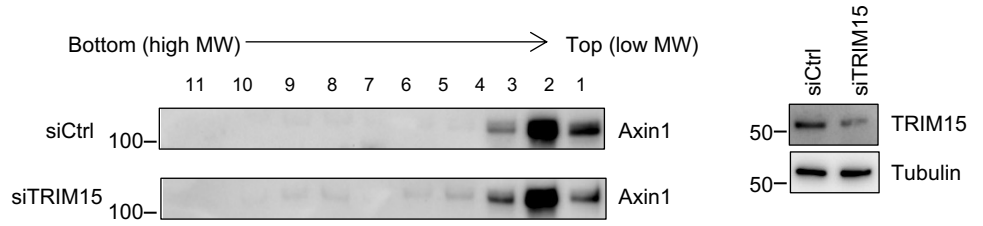**c**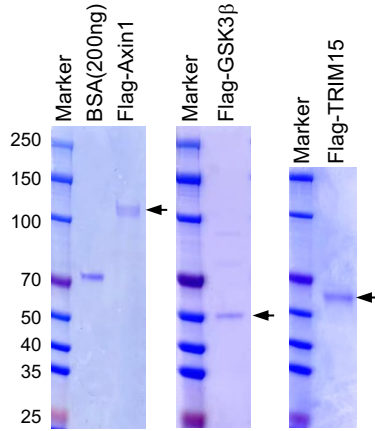**e**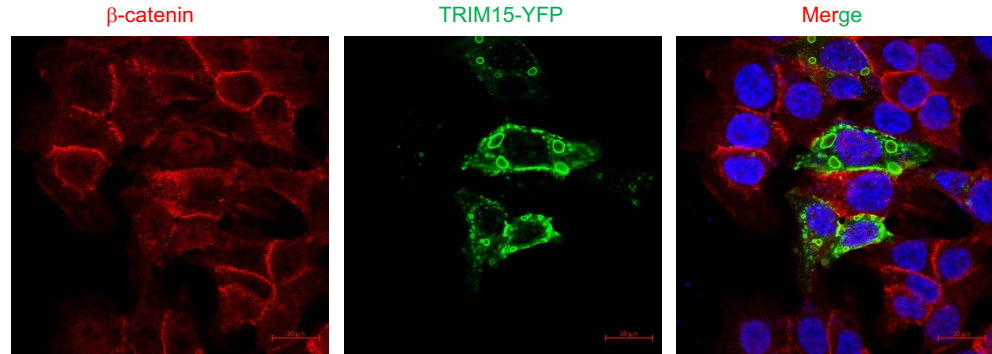**d**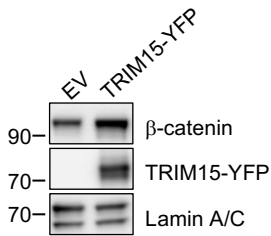**f**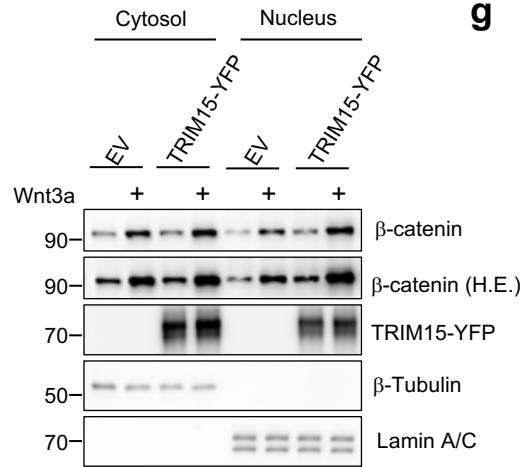**g**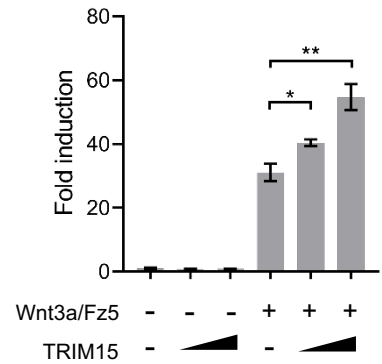

Supplement: Supplementary file 4 — Extended Data Figure 3 [file 41419_2025_8400_MOESM4_ESM.pdf]

**a**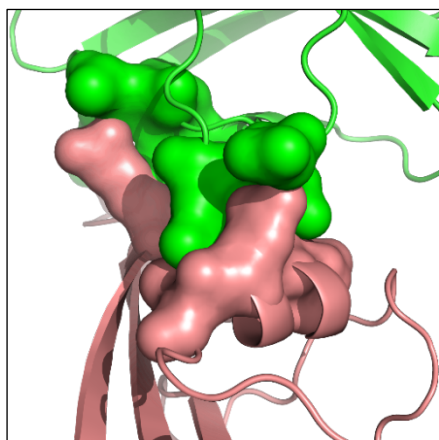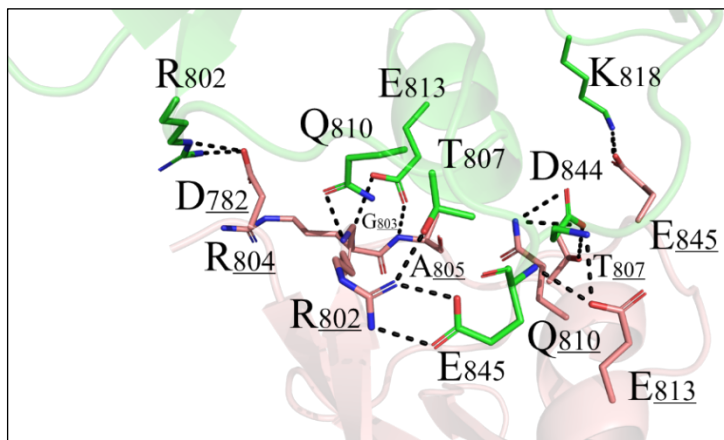**b**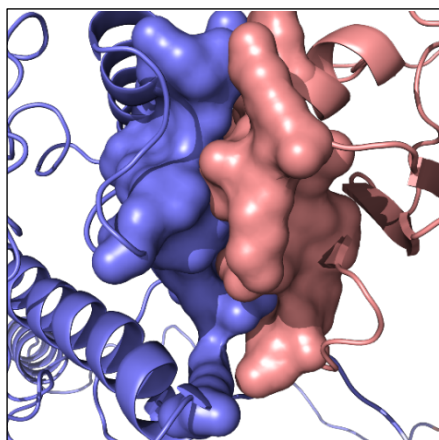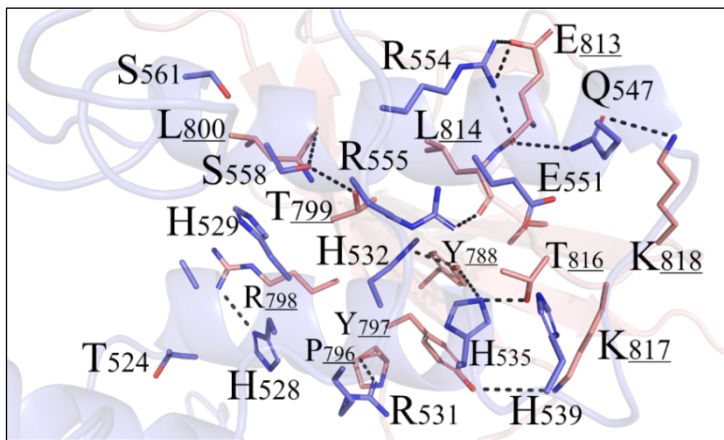**c**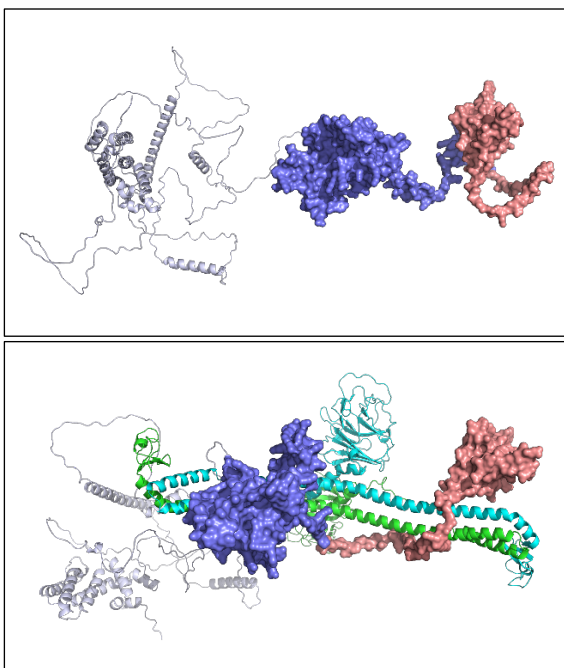**d**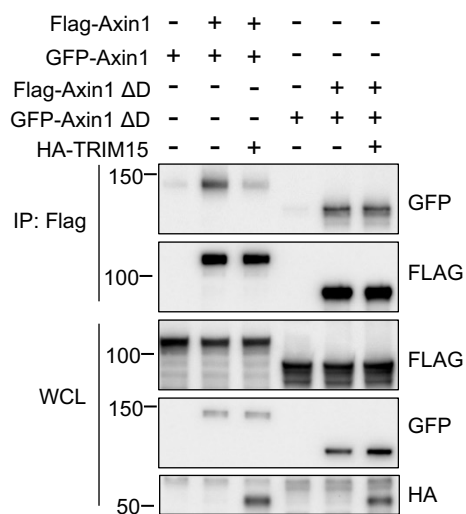

Supplement: Supplementary file 5 — Extended Data Figure 4 [file 41419_2025_8400_MOESM5_ESM.pdf]

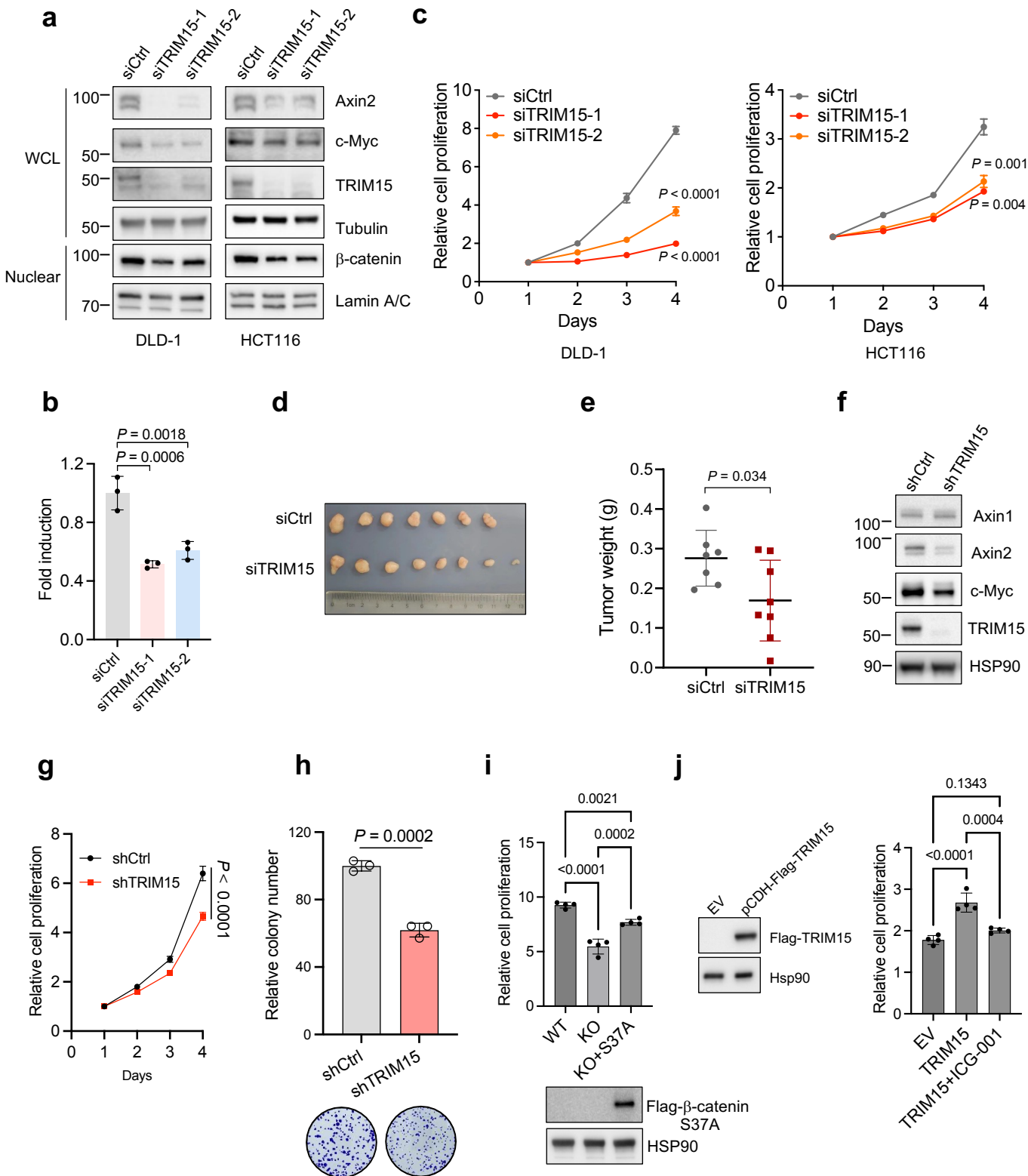

Supplement: Supplementary file 6 — Extended Data Figure 5 [file 41419_2025_8400_MOESM6_ESM.pdf]

**a**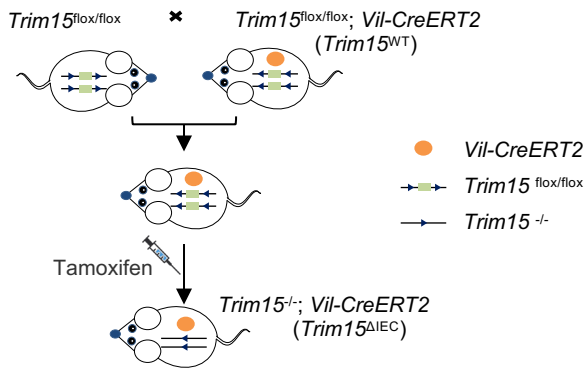**b**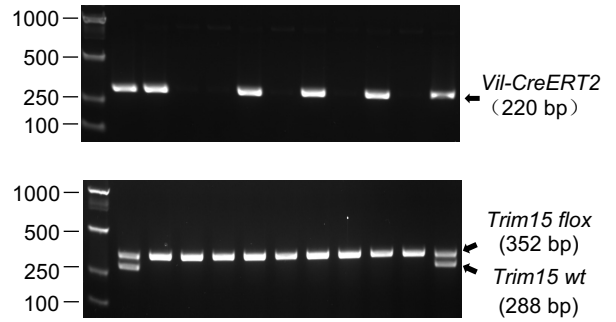**c**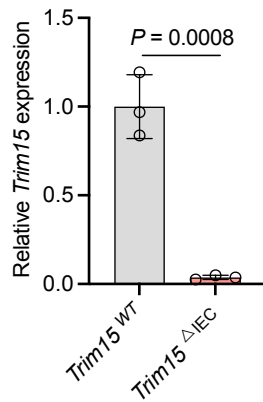**d**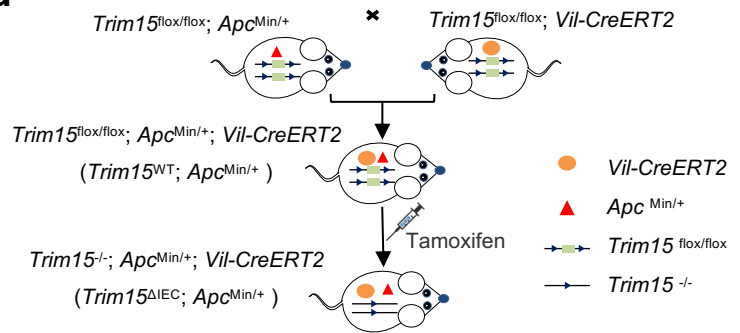**e**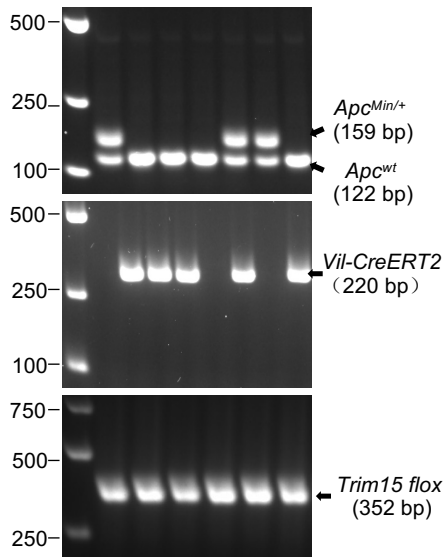**g**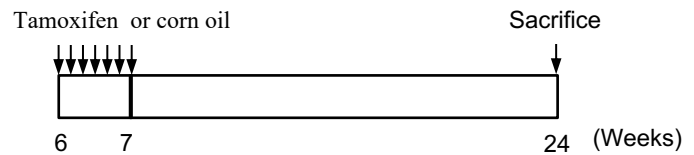**f**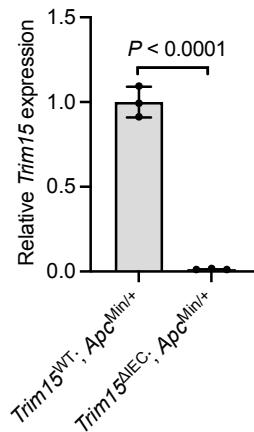**h**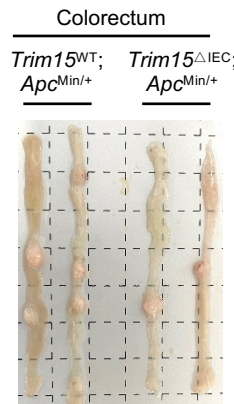**i**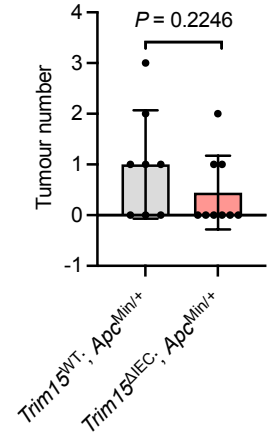

Supplement: Supplementary file 7 — Extended Data Figure 6 [file 41419_2025_8400_MOESM7_ESM.pdf]

**a**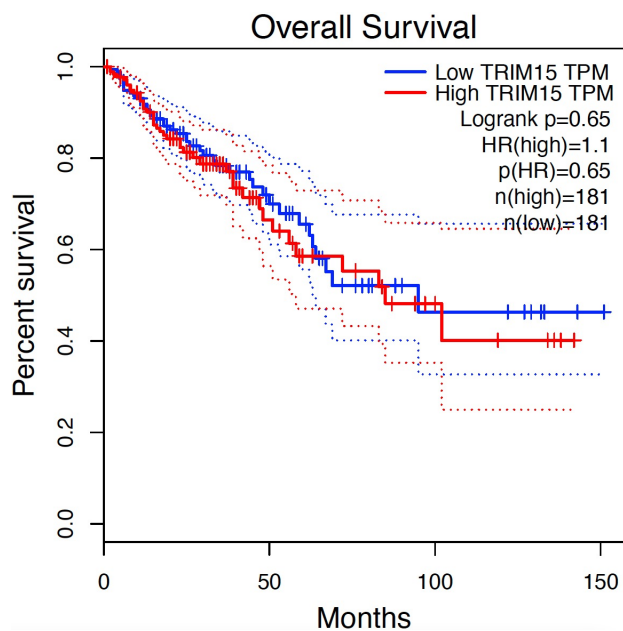**b**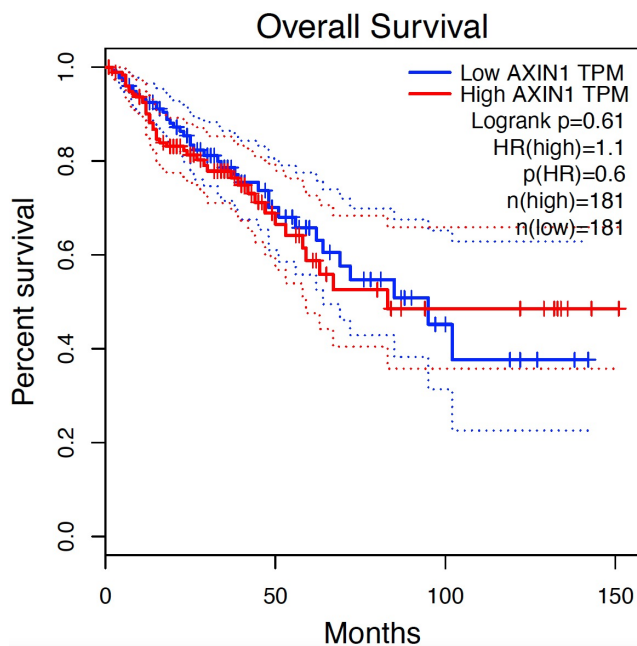**c**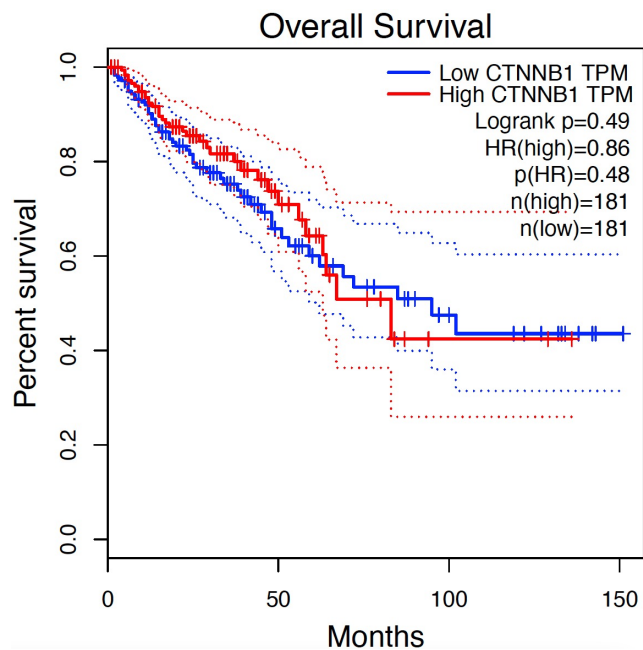**d**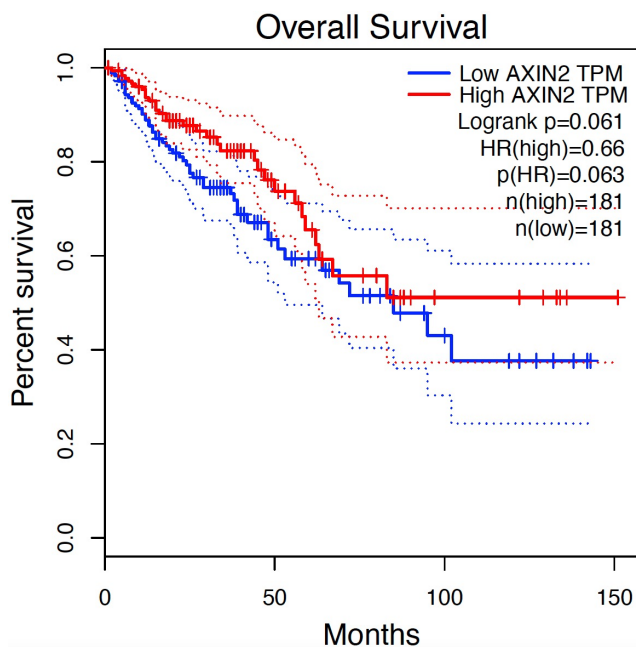

Supplement: Supplementary file 9 — Extended Data Figure 8 [file 41419_2025_8400_MOESM9_ESM.pdf]

**a**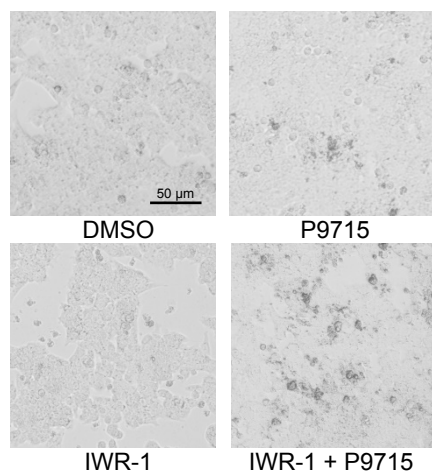**b**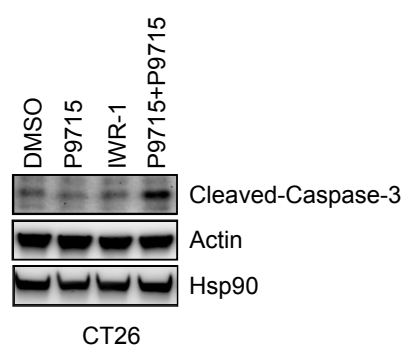**c**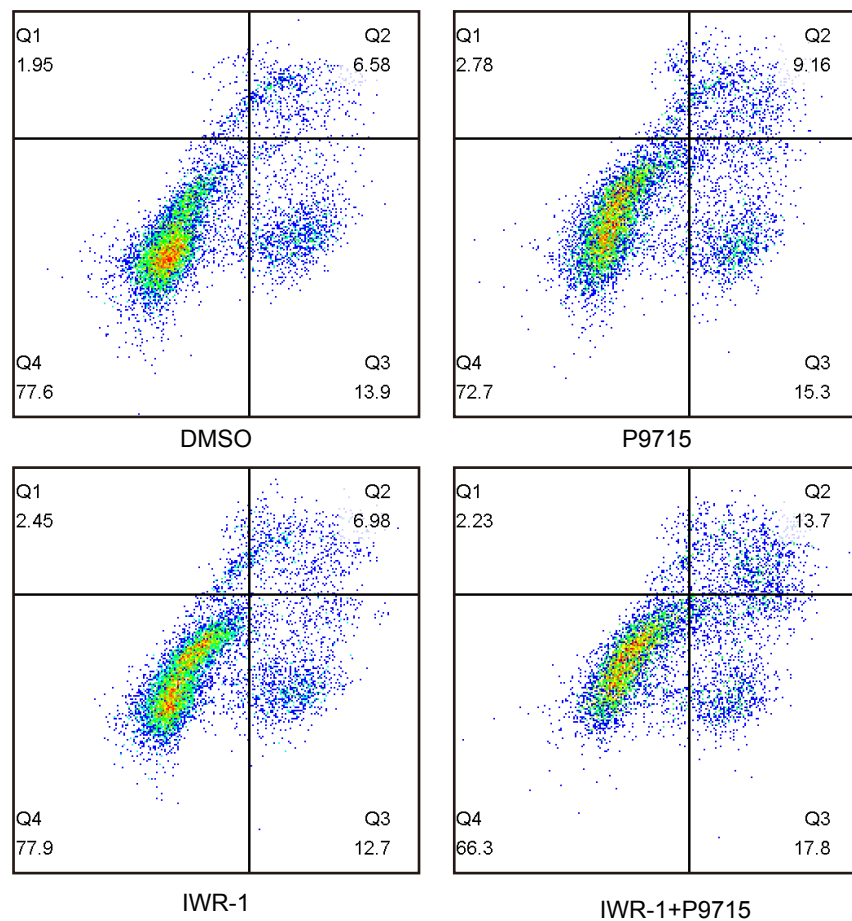

Supplement: Supplementary file 10 — Extended Data Figure 9 [file 41419_2025_8400_MOESM10_ESM.pdf]
