# Supplementary material for: Targeting TRIM15-mediated Axin1 depolymerization suppresses Wnt signaling and inhibits colorectal cancer growth
Source: Cell Death Dis. 2025 Dec 29;17(1):152. doi: 10.1038/s41419-025-08400-7 (PMC12859064; doi:10.1038/s41419-025-08400-7)
Supplement: Supplementary file 8 — Extended Data Figure 7 [file 41419_2025_8400_MOESM8_ESM.pdf]

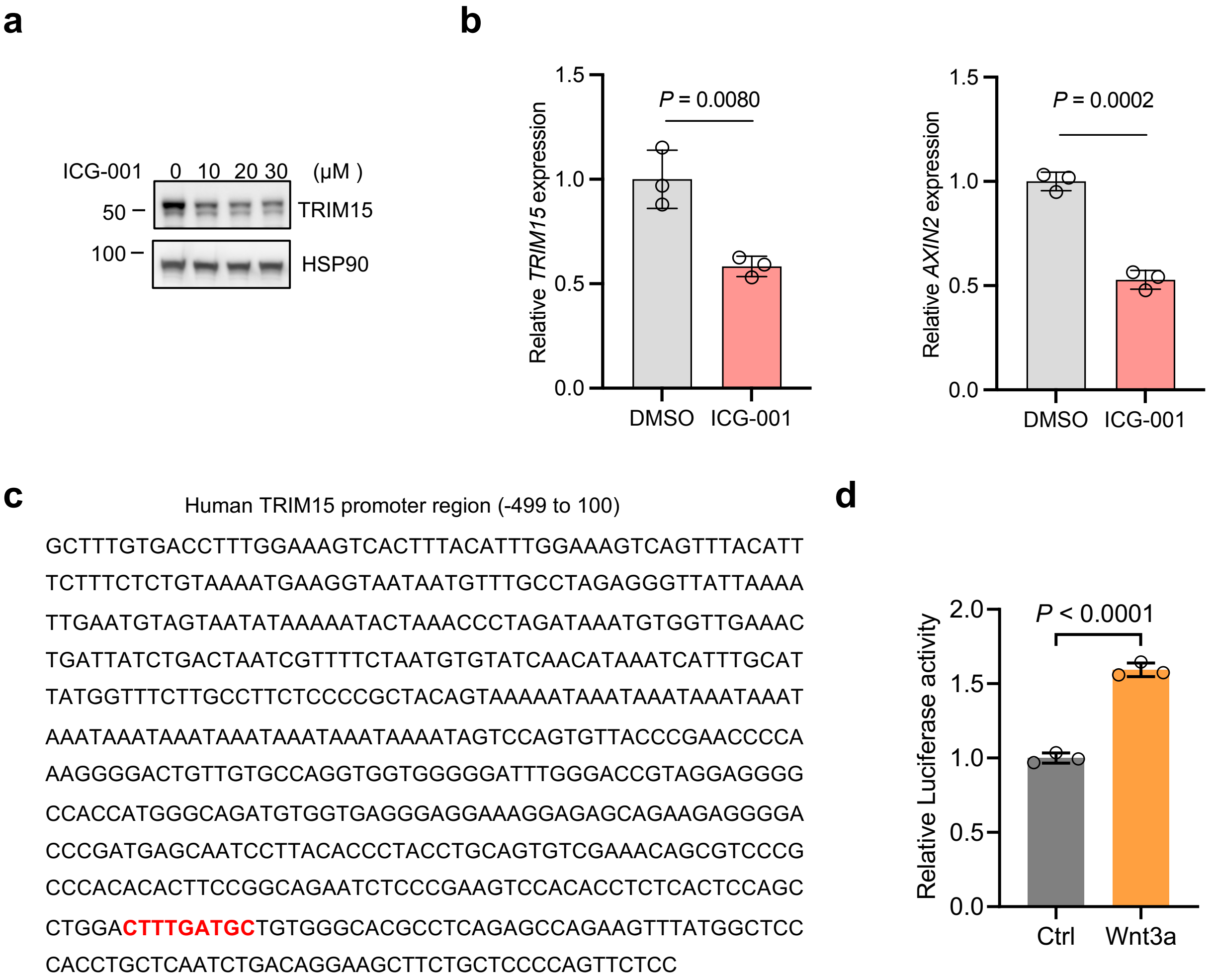

a
ICG-001 0 10 20 30 (μM)
50 —
100 —
TRIM15
HSP90
b
1.5
1.0
0.5
0.0
Relative TRIM15 expression
P = 0.0080
DMSO
ICG-001
Relative AXIN2 expression
P = 0.0002
DMSO
ICG-001
c
Human TRIM15 promoter region (-499 to 100)
GCTTTGTGACCTTTGGAAAGTCACTTTACATTTGGAAAGTCAGTTTACATT
TCTTTCTCTGTAAAATGAAGGTAATAATGTTTGCCTAGAGGGTTATTAAAA
TTGAATGTAGTAATATAAAAATACTAAACCCTAGATAAATGTGGTTGAAAC
TGATTATCTGACTAATCGTTTTCTAATGTGTATCAACATAAATCATTTGCAT
TATGGTTTCTTGCCTTCTCCCCGCTACAGTAAAAATAAATAAATAAATAAAT
AAATAAATAAATAAATAAATAAATAAAATAGTCCAGTGTTACCCGAACCCCA
AAGGGGACTGTTGTGCCAGGTGGTGGGGGATTTGGGACCGTAGGAGGGG
CCACCATGGGCAGATGTGGTGAGGGAGGAAAGGAGAGCAGAAGAGGGGA
CCCGATGAGCAATCCTTACACCCTACCTGCAGTGTCGAAACAGCGTCCCG
CCCACACACTTCCGGCAGAATCTCCCGAAGTCCACACCTCTCACTCCAGC
CTGGACTTTGATGCTGTGGGCACGCCTCAGAGCCAGAAGTTTATGGCTCC
CACCTGCTCAATCTGACAGGAAGCTTCTGCTCCCCAGTTCTCC
d
2.0
1.5
1.0
0.5
0.0
Relative Luciferase activity
P < 0.0001
Ctrl
Wnt3a
